# Brain patterns linked to neuropsychiatric genetic risk mirror those seen in disease

**DOI:** 10.1162/IMAG.a.1152

**Published:** 2026-03-10

**Authors:** Chun-Ju Chou, Elisabetta C. del Re, Hao Wang, Kareem Hamada, Xiaoguang Tian, Olena Iakunchykova, Yunpeng Wang, Mark Fiecas, Chi-Hua Chen

**Affiliations:** Department of Radiology, University of California, San Diego, CA, United States; Department of Bioengineering, University of California, San Diego, CA, United States; Department of Psychiatry, Harvard Medical School, Boston, MA, United States; Department of Psychology, University of Oslo, Oslo, Norway; Division of Biostatistics, University of Minnesota School of Public Health, Minneapolis, MN, United States

**Keywords:** imaging genetics, brain morphology, polygenic risk score, MRI, neuropsychiatric disorders, Mendelian randomization

## Abstract

Analyzing brain morphological changes across individuals with varying genetic risk scores may reveal patterns of brain alterations from health to disease. This study investigates gray matter structural alterations in individuals with clinical diagnoses compared with those with genetic risk alone. UK Biobank MRI and genotypes (N = 34,720) were used to derive brain measures and polygenic risk scores, creating genetic risk brain maps for 14 neuropsychiatric disorders. Eight disorders from ENIGMA were used to construct disease brain maps. Brain maps of genetic risk and clinical diagnosis show overall alignment for ADHD, schizophrenia, bipolar disorder, and autism. Other conditions, including Alzheimer’s disease, show specific brain regions linked to genetic risk aligning with established patient patterns. Incomplete data for some conditions limit analyses. ADHD and PTSD polygenic burden was associated with smaller global brain sizes, while Parkinson’s disease was linked to larger brain volume. Mendelian randomization analyses revealed unidirectional relationships where the brain influences ADHD and Parkinson’s disease, while a bidirectional causal association was observed for schizophrenia. Focusing on schizophrenia and bipolar disorder, we found that individuals with high genetic risk combined with smaller brain structures were more likely to have these diagnoses. Overall, the study demonstrates marked similarities in brain changes between clinical diagnoses and genetic risk for several disorders, albeit with mild effect sizes in the latter. These findings underscore the importance of genetic risk in influencing brain anatomy and the progression of neuropsychiatric disorders.

## 1 Introduction

The development and function of the cerebral cortex are often affected in neuropsychiatric disorders. To better understand the pathogenesis of these disorders, intermediate phenotypes have been used as a way to investigate the relationship between genetics and disease. Intermediate phenotypes, also known as endophenotypes, are measurable traits linked to a particular disorder. Brain structure remains a promising intermediate phenotype for neuropsychiatric disorders due to its high heritability (Blokland et al., 2024; Eyler et al., 2011; Lenroot et al., 2009; Makowski et al., 2022; Peper et al., 2007; Rimol et al., 2010; Schmitt et al., 2019, 2020; van der Meer et al., 2020), and its association with these diseases (del Re et al., 2023; Thompson et al., 2020). Neuropsychiatric disorders themselves are heritable, with moderate to high heritability values based on twin studies. For example, bipolar disorder (BPD), schizophrenia (SZ), and attention-deficit hyperactivity disorder (ADHD) have heritability values of ~ 75–80%, while major depression disorder (MDD) displays values of ~ 40% (Bray & O’Donovan, 2019; McGuffin et al., 2003; Rietveld et al., 2003; Sullivan et al., 2000, 2003).

Meta-analyses conducted by the ENIGMA consortium, which combine studies to derive large samples of individuals affected by various neuropsychiatric disorders (Bray & O’Donovan, 2019; McGuffin et al., 2003; Rietveld et al., 2003; Sullivan et al., 2000, 2003), have provided valuable insights into brain morphological changes in disease states based on case–control comparison (N range: ~700 to ~4500, see Table 1) (Boedhoe et al., 2017; Hibar et al., 2018; Hoogman et al., 2017; Schmaal et al., 2017; Sun et al., 2022; Thompson et al., 2014; van Erp et al., 2016; van Rooij et al., 2018; Whelan et al., 2018). For instance, SZ is characterized by enlarged lateral ventricles and reduced hippocampus (van Erp et al., 2016), a pattern shared with early-onset MDD (van Erp et al., 2016) and BPD (Hibar et al., 2016). Here, our focus is on investigating such changes in a large community-based volunteer cohort, composed mostly of individuals without diagnosed neuropsychiatric disorders with varying levels of genetic risks and assessing whether any similar patterns exist between diagnoses and risks. Analyzing brain morphological changes across individuals with genetic risk scores enables an exploration of how brain morphology varies along the dimension of genetic liability. This rationale aligns with a viewpoint interpreting neuropsychiatric diseases as being the extremes of quantitative dimensions (e.g., variations in brain structure) influenced by polygenic predispositions (Plomin et al., 2009). An advantage of studying a cohort largely without diagnosed neuropsychiatric disorders is that findings are less confounded by factors that are difficult to control for in cases, such as medication status and duration of illness.

**Table 1.** Overview of genome-wide association studies (GWAS) for neuropsychiatric diseases and corresponding cortical magnetic resonance imaging (MRI) studies.

Diseases	GWAS (N cases/controls)	MRI (N cases/controls)
ADHD & ADHD age-specific	Demontis et al., 2023 (38,691/186,843) Rajagopal et al., 2022 Childhood-15,338/45,398 Adulthood-6961/38,303	Hoogman et al., 2019 (2246/1934) <15 years old: (1081/1048) ≥22 years old: (733/539)
ASD	Grove et al., 2019 (18,381/27,969)	Van Rooij et al., 2018 (1571/1651)
BPD	Mullins et al., 2021 (41,917/371,549)	Hibar et al., 2018 <25 years old: (411/1035) ≥25 years old: (1837/2582)
MDD	Wray et al., 2018 (59,851/113,154)	Schmaal et al., 2017 <21 years old: (294/237) ≥21 years old: (1911/7663)
OCD	IOCDF-GC and OCGAS, 2018 (2699/7030)	Boedhoe et al., 2017 <18 years old: (407/324) ≥18 years old: (1498/1436)
PTSD	Nievergelt et al., 2019 (20,329/124,440)	Sun et al., 2022 (1348/2066)
SZ	Trubetskoy et al., 2022 (53,886/77,258)	Van Erp et al., 2018 (4474/5098)
TS	Yu et al., 2019 (4819/9488)	-
AD	Kunkle et al., 2019 (17,008/37,514)	-
ALS	van Rheenen et al., 2016 (12,577/23,475)	-
EP	ILAE, 2018 (15,212/29,677)	Whelan et al., 2018 (2149/1727)
FTD	Ferrari et al., 2014 (2154/4308)	-
PD	Nalls et al., 2014 (13,708/95,282)	Laansma et al., 2021 (2357/1182)
AN	Watson et al., 2019 (16,992/55,525)	Walton et al., 2022 (685/963(Female))

For BPD, MDD, and OCD, only age-specific subgroups are provided, without a combined total group. Brain maps for PTSD and AN were not generated in Figure 2 due to data limitations: PTSD data are available only in the Destrieux atlas, and AN lacks GWAS excluding UKB samples.

ADHD: attention-deficit/hyperactivity disorder, ASD: autism spectrum disorder, BPD: bipolar disorder, MDD: major depressive disorder, OCD: obsessive-compulsive disorder, PTSD: post-traumatic stress disorder, SZ: schizophrenia, TS: Tourette syndrome, AD: Alzheimer’s disease, ALS: amyotrophic lateral sclerosis, EP: epilepsy, FTD: frontotemporal dementia, PD: Parkinson’s disease, AN: anorexia nervosa, IOCDF-GC: International Obsessive Compulsive Disorder Foundation Genetics Collaborative, OCGAS: OCD Collaborative Genetics Association Studies, ILAE: International League Against Epilepsy Consortium.

We calculated genetic risks or liability using polygenic risk scores (PRS) from approximately 34,000 participants in the UK Biobank (UKB), representing a large community-based volunteer cohort. The goal was to determine correlations between brain measures and PRS of 14 neuropsychiatric disorders, detailed in Table 1. Previous PRS–brain association studies in UKB have mostly focused on specific diseases, with fewer performing cross-disorder analyses (Abbasi et al., 2022; Guo et al., 2022; He et al., 2023; Korbmacher et al., 2024; Reus et al., 2017; Rodrigue et al., 2023; X. Zhu et al., 2021). These include recent investigations of Parkinson’s disease (Abbasi et al., 2022) and SZ (Alnæs et al., 2019; Franke et al., 2016; Grama et al., 2020; Qi et al., 2022; Stauffer et al., 2021; X. Zhu et al., 2021). White matter diffusion measures have also been explored across disorders (Korbmacher et al., 2024). As genetic risk–brain associations do not imply causation, recent studies have also incorporated Mendelian Randomization (Guo et al., 2022; Stauffer et al., 2021; Walton et al., 2019). Our cross-disorder approach enables the comparison of brain morphometry across diseases. Traditionally, studies focus on single diseases with varying methodologies and sample sizes (often smaller in earlier studies using earlier-release data), making cross-study comparisons challenging. By analyzing both regional cortical and subcortical structures, this study facilitates direct morphometry comparisons within and across disorders (Guo et al., 2022; X. Zhu et al., 2021).

This study hypothesizes that neuropsychiatric risk genes influence brain endophenotypes even in asymptomatic individuals. The goals are to examine associations between 14 neuropsychiatric genetic risks and mostly gray matter morphology, employing Mendelian randomization to explore bidirectional causality between brain structure and disease. The study also compares spatial patterns of brain alterations in genetic predisposition and disease states, assessing regional concordance. Finally, it investigates whether heightened genetic risk corresponds to diagnosis by comparing genetic liability and brain structure distributions between healthy individuals and those with psychiatric conditions.

## 2 Methods

### 2.1 Sample

Genomic, imaging, and demographic data were extracted from the UKB population cohort, under accession number 27412 (Bycroft et al., 2018; Elliott et al., 2018; Miller et al., 2016; Sudlow et al., 2015). Quality control (QC) of imaging and demographic data were detailed previously (Makowski et al., 2022). In brief, we excluded individuals with bad structural scan quality, based on the Euler numbers more than 3 standard deviations lower than the scanner site mean (N = 594) (Dale et al., 1999). We removed related individuals before the association testing. Using GCTA (Yang et al., 2011), we calculated the pairwise genetic relationship matrix (GRM) based on genome-wide autosomal variants and removed one related individual from pairs (N = 859) with an estimated GRM greater than 0.1, which indicates relatedness closer than third cousins. Our final sample included 33,861 participants of genetically inferred European ancestry based on UKB genetic ancestry grouping data (age range: 45.13–81.83 years, male–female ratio of 0.9). We did not exclude participants with medical diagnoses. While disease prevalence in the UKB is generally comparable with the general population, it tends to be lower for certain conditions such as cancer due to a volunteer selection bias (Fry et al., 2017).

This study was conducted using data from UKB approved by the institutional review board (IRB) of UCSD. All analyses were performed on de-identified data provided by UKB. Summary-level neuroimaging results from the ENIGMA consortium used for comparative analyses were obtained from published sources and involved no access to individual-level identifiable data. No new participants were recruited for this study. As such, no additional informed consent was required for these analyses.

### 2.2 Genotype data

We used UKB Version 3 release of imputed genotype data and removed individuals with more than 10% missingness, as well as single nucleotide polymorphisms (SNPs) with more than 5% missingness, failing the Hardy–Weinberg equilibrium test at p = 10^-6^ or with minor allele frequencies (MAF) below 0.01 (Makowski et al., 2022).

### 2.3 MRI data and atlases

T1-weighted MRI scans were collected from three scanning sites throughout the UK, all on identical Siemens Skyra 3T scanners (Miller et al., 2016). The standard “recon-all-all” processing pipeline of Freesurfer v5.3 was applied to perform automated surface-based morphometry segmentation (Fischl et al., 2002). The latest UKB Freesurfer v6.0 derivatives were not incorporated in the present study to ensure consistency with prior analyses conducted using v5.3.

For cortical phenotypes, we adopted two genetically informed atlases, including 12 regions for surface area (SA) and 12 for cortical thickness (CT), and 2 global measures of total surface area and mean thickness. Our group previously developed these atlases using a data-driven fuzzy clustering technique to identify parcels of the human cortex that are maximally genetically correlated (Chen et al., 2012). The subcortical structures were parcellated based on the widely used *Aseg* atlas (Fischl et al., 2002), including volumes of 16 regions and 1 global measure of estimated intracranial volume (ICV). We combined measures of each phenotype across both hemispheres, given the largely bilateral symmetry of genetic patterning demonstrated previously (Chen et al., 2011; Makowski et al., 2022).

### 2.4 Polygenic risk score

We calculated the PRS of the above neuropsychiatric phenotypes by PRS-CS, among the UKB samples (Ge et al., 2019). Publicly available summary data were downloaded (Supplementary Table S1). Quality control steps include the removal of SNPs with low imputation information score (<0.8) or minor allele frequency (<0.01), and duplicated or ambiguous SNPs. In neuropsychiatric GWAS involving UKB samples, we opted for their subsample GWAS data, after excluding UKB samples, to ensure no significant sample overlaps between the source and target datasets in generating PRS. Given the UKB cohort’s predominance of European ancestry, we restricted our analysis to GWAS summary statistics from individuals of European descent to reduce bias.

### 2.5 PRS–brain morphology association analysis

Prior to association analysis, we regressed out age, sex, scanner site, a proxy of scan quality (FreeSurfer’s Euler number) (Dale et al., 1999), and the first 10 genetic principal components from each neuroimaging-derived measurement. Subsequently, we applied a rank-based inverse normal transformation to the residuals of each measure, ensuring normally distributed input to each association testing. Linear regression models were applied using the R lm function to conduct association testing. These models aimed to investigate the relationships between standardized PRS as the independent variables and brain morphometric measures as the dependent variables, considering each pair of diseases and brain regions individually.

### 2.6 Determining effective number of independent phenotypes

To consider the correlation between phenotypes, we applied matSpD to determine the effective number of independent phenotypes (*t*_e_) (Li et al., 2012), using correlation matrices of cortical and subcortical measures. Statistical significance was determined using the Bonferroni correction for multiple comparisons, with a significance threshold set at p < 0.05/*t*_e_ (see Supplementary Methods).

### 2.7 Comparative analysis contrasting brain maps associated with disease states and genetic risk

The genetic risk brain maps in the right panel of Figure 2 use individual-level data from UKB. The diagnostic brain maps in the left panel rely on summary-level data from ENIGMA. The ENIGMA studies used the typical Desikan–Killiany (DK) brain atlas. Cohen’s *d* effect size of every DK brain region was estimated from the t-value for the group difference. All neuroimaging analyses were surface based by FreeSurfer (Dale et al., 1999). Our brain regions were converted to the DK atlas to align with the ENIGMA data. To compare with ENIGMA case–control studies, we dichotomized continuous PRS into high- and low-risk groups based on the median (Thompson et al., 2014), and subsequently calculated Cohen’s d. Additional comparative tests were implemented and detailed (Supplementary Methods).

We obtained the summary-level data from the ENIGMA TOOLBOX, a neuroimaging repository of meta-analytical case–control comparisons of cortical or subcortical structure traits for seven psychiatric disorders (ADHD, ASD, BPD, EP, MDD, OCD, and SZ). The PD data were sourced from Laansma et al., a study within the ENIGMA-Parkinson’s project (Laansma et al., 2021). For BPD, MDD, and OCD, only age-specific subgroups (young and adult groups) are provided, without a combined total group. For the other disorders, only combined total groups are available, except for ADHD, which includes both total and age-specific subgroups (see Table 1 and Supplementary Table S8). The adult subgroups were chosen for BPD, MDD, and OCD due to their larger sample sizes. For ADHD, the pediatric subgroup was selected because of its larger sample size and the condition’s predominance in childhood diagnoses. Besides these main results, we included comparison analyses for subcortical regions (Supplementary Fig. S5) and across all subgroups (Supplementary Figs. S6 and S7). Note that not all disorders have both global adjusted and unadjusted measures in ENIGMA studies, we aligned our analysis with the ENIGMA data availability.

### 2.8 Mendelian randomization

We conducted Mendelian randomization (MR) analysis to explore the causal effects of the global brain morphometric measures on neuropsychiatric disorders, as well as the reversed causal effects from disorders to the brain. MR analyses were performed through various methods such as weighted median, inverse-variance weighted **(**IVW), Egger regression, weighted mode, and generalized summary-based MR (GSMR) utilizing the TwoSampleMR and gsmr v1.1.0 R packages (Z. Zhu et al., 2018). The selection of the instrumental variables (IVs), the QC procedure, and testing the robustness via the MR sensitivity analysis are described in the Supplementary Methods.

### 2.9 Comparing scatterplots and density distributions of PRS and brain structure between healthy and diagnosed individuals

To ascertain whether heightened PRS corresponds to clinical diagnosis, we extracted patient samples from the UKB data. We specifically focused on SZ and BPD to explore this analysis, prioritizing them due to significant findings in Figure 2 and the limited sample sizes for some other disorders in UKB. We examined clinical diagnosis details for these conditions (see Supplementary Methods). Particularly, characteristics of UKB participants with SZ have been thoroughly studied (Legge et al., 2024).

All the UKB samples were plotted on a scatterplot of brain structure variation against PRS, where brain structure and PRS values were standardized. Only global brain measures were assessed, making this comparison independent of any specific cortical atlas. The hypothesis was that both high genetic liability and smaller brain sizes are risk factors for psychiatric conditions. The null hypothesis posits that the proportion of patients exhibiting high PRS and low brain measures is equal to 0.25. Binomial tests were conducted to assess that the diagnosed individuals with high PRS and reduced global brain measurements significantly differ from the random chance of 25% (Supplementary Methods).

## 3 Results

### 3.1 Cortical and subcortical morphology associated with polygenic risks of neuropsychiatric phenotypes

**Fig. 1.**
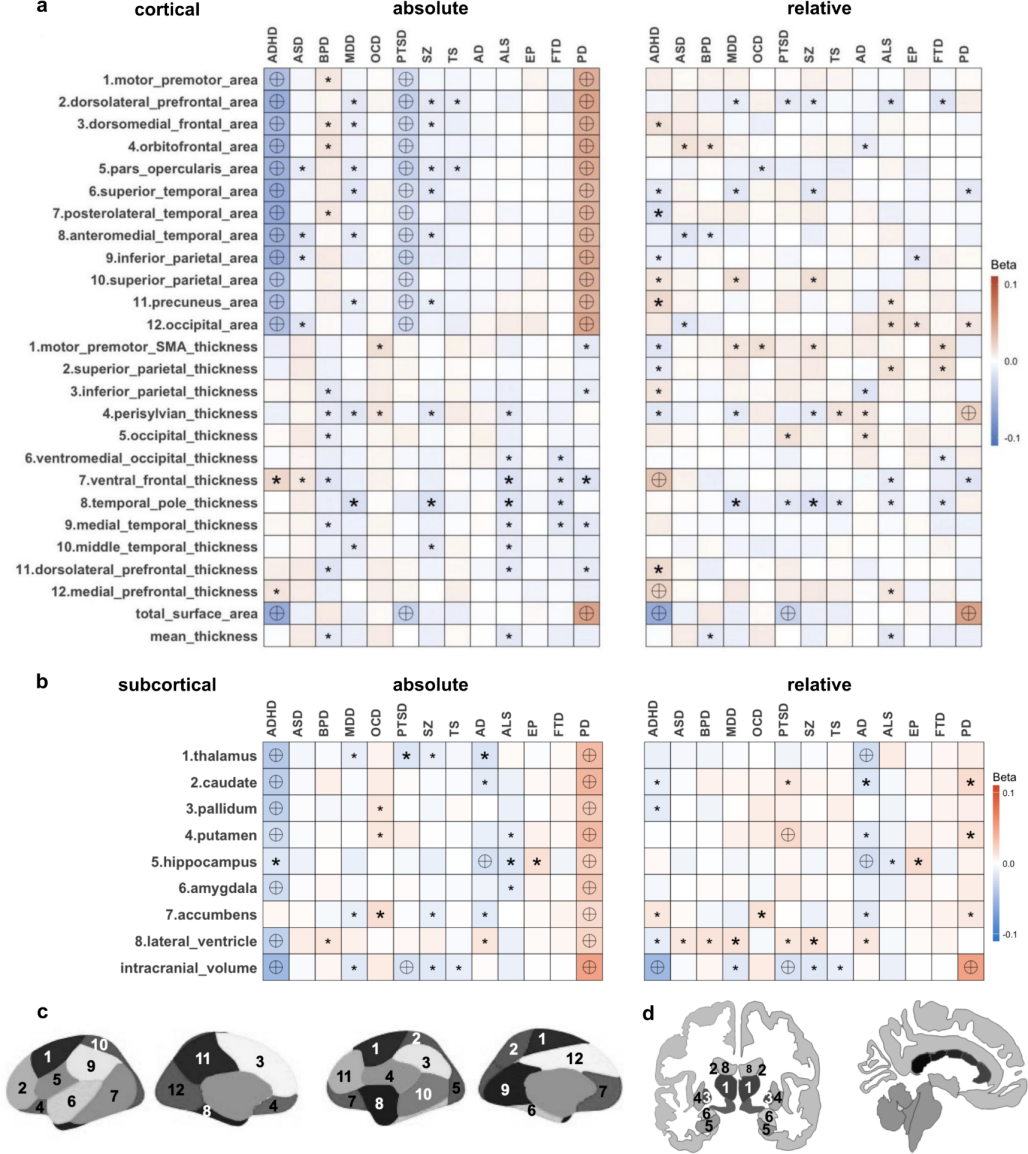
Heatmap of associations between polygenic risk scores and cortical surface area, cortical thickness, and subsetted subcortical volume. (a) Cortical measurements for absolute regions (left), and cortical measurements for relative regions (right). Global measures are presented twice at the bottom of both top heatmaps. (b) Subcortical measurements for absolute regions (left), and subcortical measurements for relative regions (right). (c) Schematic brain cortical regions (Chen genetic atlas) with numbers as a reference in panel (a) (Chen et al., 2012). (d) Schematic brain subcortical regions as (aseg atlas) with numbers as a reference in panel (b). Boxes are colored according to the beta coefficients of linear regression models. Various stringency thresholds are used, including nominal significance (p < 0.05, denoted by small asterisks), Bonferroni-corrected threshold for individual diseases (disease-wise significance denoted by large asterisks, cortical: p < 2.3 × 10^-3^, subcortical: p < 7.2 × 10^-3^) and Bonferroni correction for total comparisons (study-wise significance represented by encircled crosses, cortical: p < 1.7 × 10^-4^, 22 regions × 13 diseases, subcortical: p < 5.5 × 10^-4^, 7 regions × 13 diseases). This multiple-threshold approach enables us to compare with published studies focusing on single diseases.

PRS scores were normally distributed (Supplementary Fig. S1). Out of the 14 neuropsychiatric diseases, AN was later excluded because the GWAS summary data without the UKB sample were not available. We introduced the terms absolute regional measures (without global brain size adjustment; Fig. 1a, left; Supplementary Table S2) and relative regional measures (with global brain size adjustments; Fig. 1a, right; Supplementary Table S3) to present the results. By presenting both, we aimed at excluding the influence of brain size on regional measures.

For absolute regional measures, we observed that increased SA exhibited significant associations with PRS for PD, while decreased SA showed associations with PRS for ADHD and PTSD, across most regions and the total area. Our analysis revealed less robust evidence of an association between PRS and CT. No other significant associations were identified at the study-wise threshold, but a few reached significance at the disease-wise threshold. For relative regional associations of SA and CT, there was a general attenuation in significant associations.

We included eight commonly used and validated subcortical regions (Liem et al., 2015), consistent with ENIGMA studies: thalamus, caudate, pallidum, putamen, hippocampus, amygdala, accumbens, and lateral ventricles. The remaining FreeSurfer aseg regions are reported in the SI. In the absolute regional measures (Fig. 1b, left; Supplementary Table S4), significant associations were evident at the study-wise threshold: smaller subcortical regions and ICV were associated with PRS for ADHD, whereas the opposite trend was observed for PD (Supplementary Fig. 2). PRS for AD was associated with reductions in the hippocampus. In the relative regional measures, most of the associations with PD disappeared, suggesting that global measures play a significant role as a common driving factor across multiple regions in this context (Fig. 1b, right; Supplementary Table S5). Additional exploratory sex-stratified analyses are provided in Suppleemntary Figure 4, Supplementary Tables S10 and S11.

### 3.2 Similarities between brain patterns associated with neuropsychiatric diagnoses and those associated with genetic risk

**Fig. 2.**
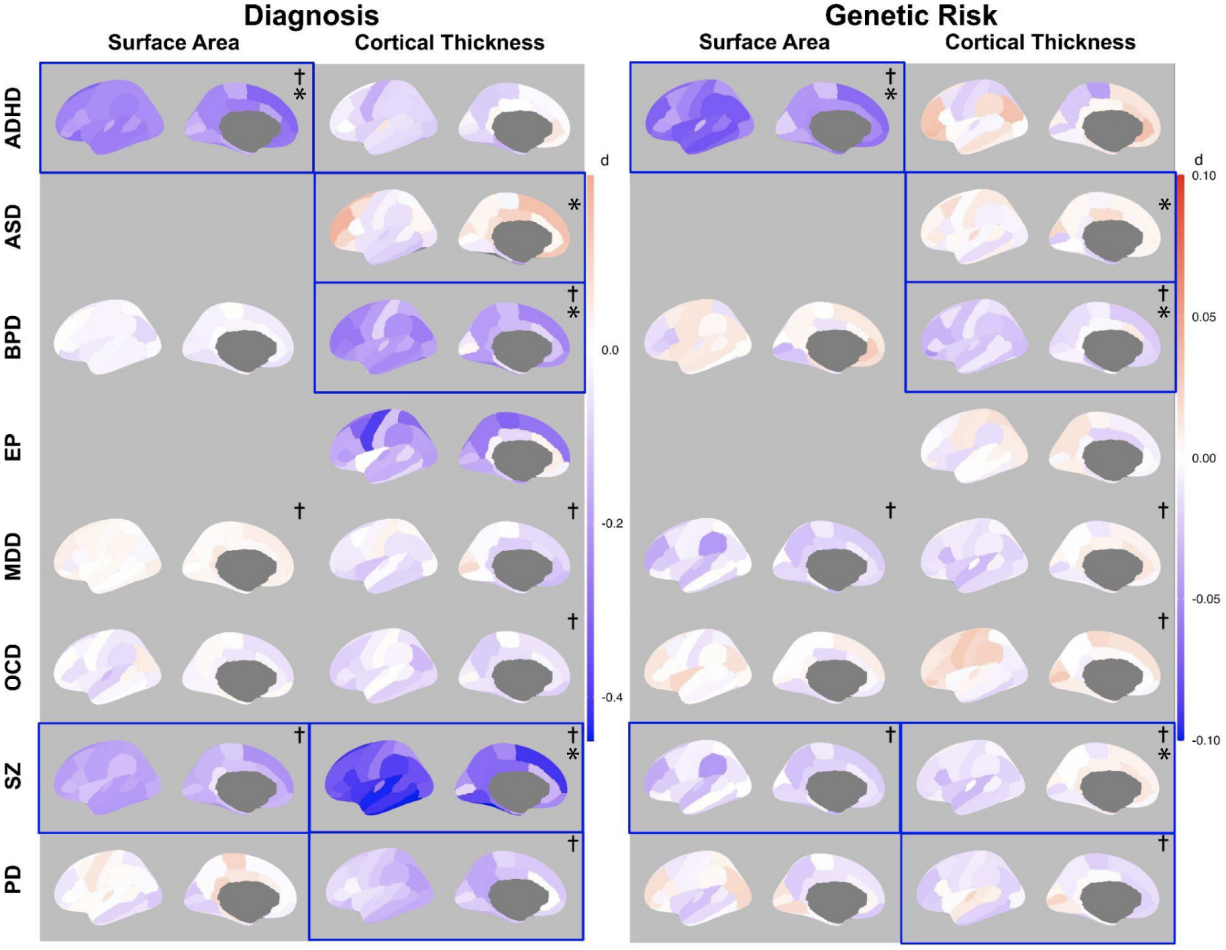
Cohen’s d brain maps illustrate the differences in cortical measurements between patients versus healthy controls, as well as high- and low-risk scores for neuropsychiatric disorders. On the left panel, the brain maps present the spatial layouts of case–control differences in SA and CT as Cohen’s d values. These were extracted from published studies, involving patient cohorts with eight neuropsychiatric diagnoses from the ENIGMA consortium. The brain maps are based on cohorts across different age groups: children (ADHD), adults (BPD, MDD, OCD), and total/lifespan (ASD, SZ, EP, PD). Detailed information about the group cohorts is given in section 2 and Supplementary Table S8. The right panel illustrates brain maps displaying the effect sizes of the high vs low PRS comparisons in brain morphology. Color intensity reflects the magnitude of effect size (Cohen’s d), with blue denoting a negative effect and red a positive effect. The color scale for diagnosis is (-0.45,0.2), and the genetic risk score is (-0.1,0.1). Brain maps accompanied by a cross symbol (†) indicate absolute regional measures, and asterisks (*) indicate disorders in which the correlation between the diagnosis and genetic risk brain maps survived FDR correction at p < 0.05. Brain maps outlined in blue highlight significant sign concordances.

Brain alterations linked to genetic risk were subtler but similar to those displayed by diagnosis. In Figure 2, brain alterations associated with genetic risk for ADHD, ASD, BPD, and SZ, while subtle when compared with those accompanying a formal diagnosis exhibit an overall resemblance in their underlying structural alterations. The left panel of Figure 2 displays MRI-derived Cohen’s d values to contrast cases versus controls (data from Table 1). On the right panel, Cohen’s d represents the contrast of PRS, which was priorly dichotomized into high- and low-risk groups using the median. In the analysis, we focus on the significance of the sign concordance of effect size across the brain regions, and the positive correlation results of the pairs (Table 2; Supplementary Table S8). Pairs with the nominal significance of sign concordance are outlined with blue borders.

**Table 2.** Results of comparative analysis contrasting brain maps associated with disease states and genetic risk.

	ADHD	ASD	BPD	MDD	OCD	SZ	EP	PD
Sign Concordance [%, p-value]								
SA	**100%** **5.82e-11^+^**	NA^2^	44.1% 8.04e-01	29.4% 9.95e-01^+^	47.1% 6.96e-01	**94.2%** **3.47e-08** ^+^	NA	35.3% 9.71e-01
CT	55.9% 3.04e-01	**67.7%** **2.88e-02**	**91.2%** **3.83e-07^+^**	**64.7%** **6.07e-02^+^**	47.1% 6.96e-01**^+^**	**75.5%** **4.50e-03^+^**	55.9% 3.04e-01	**70.6%** **1.22e-02^+^**
Subcortical	71.43% 2.27e-01	71.43% 2.27e-01	85.71% 6.25e-02	71.43% 2.27e-01	71.43% 2.27e-01	71.43% 2.27e-01 **^+^**	42.86% 7.73e-01	16.67% 9.84e-01
Correlation [r, p-value]^1^								
SA	**0.67** **±** **0.26** **1.00e-04^+^**	NA	-0.09 ± 0.35 6.09e-01	0.01 ± 0.35 9.74e-01**^+^**	-0.02 ± 0.35 9.32e-01	-0.16 ± 0.34 3.62e-01**^+^**	NA	-0.40 ± 0.32 1.67e-02
CT	0.34 ± 0.33 5.04e-02	**0.45** **±** **0.31** **6.50e-3**	**0.44** **±** **0.31** **1.07e-02^+^**	-0.25 ± 0.34 1.47e-01**^+^**	0.32 ± 0.33 6.62e-02**^+^**	**0.38** **±** **0.32** **2.76e-02^+^**	-0.41 ± 0.32 1.86e-02	0.10 ± 0.35 5.63e-01**^+^**
Subcortical	-0.58 ± 0.71 1.74e-01	-0.58 ± 0.72 1.66e-01	0.05 ± 0.88 9.270e-01	0.08 ± 0.87 8.37e-01	0.69 ± 0.63 8.11e-02	0.25 ± 0.85 5.78e-01**^+^**	-0.19 ± 0.86 6.86e-01	-0.92 ± 0.37 1.10e-02

Bold font denotes nominal significance.

1 Permutation-derived p-values are provided for the correlation test.

2 Data are unavailable from ENIGMA or existing literature.

+ Symbols denote absolute measures.

For SA, the sign proportion agreement for ADHD was 100% and was 94.2% for SZ, with respective p-values of 5.82 × 10^-11^ and 3.47 × 10^-8^. Additionally, ADHD exhibits a high correlation of Cohen’s d between the brain maps of diagnosis and genetic risk (r = 0.67, FDR = 7.8 × 10^-5^). For CT, a high sign concordance proportion agreement was observed for ASD (67.7%, p = 2.28 × 10^-2^), BPD (91.2%, p = 3.83 × 10^-7^), SZ (75.5%, p = 4.52 × 10^-3^), and PD (70.6%, p = 1.21 × 10^-2^). Although the spatial correlations were moderate (r ≈ 0.4), these relationships display statistically significant results after FDR correction (ASD: 1.1 × 10^-2^; BPD: 1.3 × 10^-2^; SZ: 4.6 × 10^-2^; ASD: 2.4 × 10^-2^).

### 3.3 Causal relationship between global brain morphometric measures and neuropsychiatric phenotypes

**Fig. 3.**
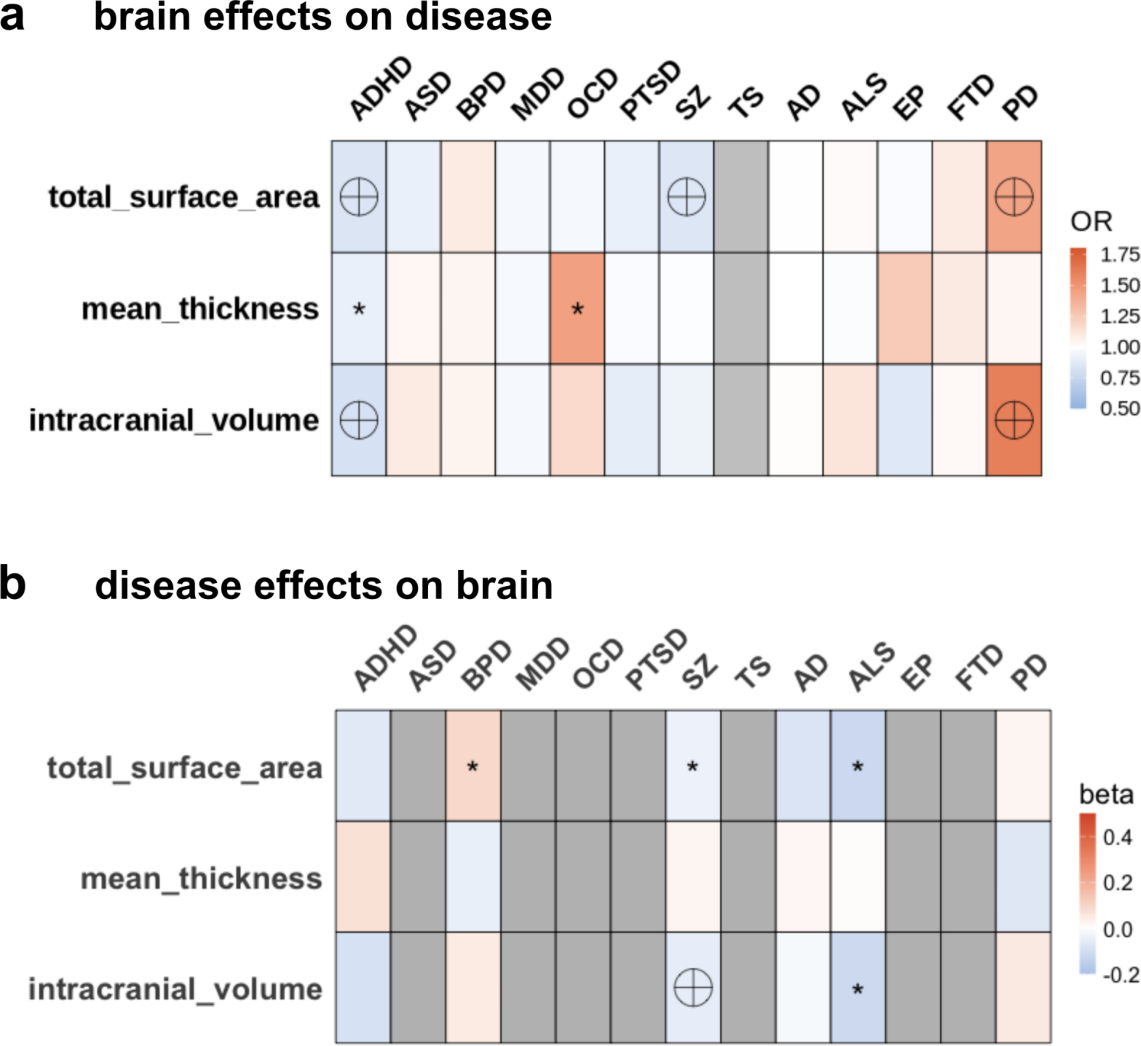
Bidirectional causal relationships between neuropsychiatric conditions and brain traits. (a) Heatmap of forward causal effects of global measures (total surface area, mean thickness and ICV) on the polygenic risk scores of 13 neuropsychiatric phenotypes. (b) Heatmap of reverse causal effects of the polygenic risk scores of 13 neuropsychiatric phenotypes on global measures (total surface area, mean thickness and ICV). Boxes are colored according to the standardized odds ratio (OR) of forward GSMR, and standardized beta of reverse GSMR. Labels denote nominal significance (p < 0.05, small asterisks), Bonferroni-corrected disease-wise significance (p < 1.6 × 10^-2^, big asterisks), and study-wise significance (p < 1.2 × 10^-3^, encircled crosses). Gray boxes represent data that were unavailable, attributed to limitations such as insufficient sample size or an inadequate number of filtered SNPs meeting the criteria for inclusion. The (a) color scale was provided as (0.5,1.8) and (b) as (-0.2,0.5).

The analysis prioritized global measures to capture broad primary patterns, and detailed examinations of individual brain regions were beyond the study’s scope. Forward GSMR analysis revealed that reduced total SA may contribute to the development of ADHD (*OR* = 0.84, 95% CI = [0.76,0.91], p = 8.1 × 10^-5^) and SZ (*OR* = 0.84, 95% CI = [0.77,0.91], p = 2 × 10^-5^), whereas increased total SA (*OR* = 1.43, 95% CI = [1.21,1.71], p = 4.1 × 10^-5^) and increased ICV (*OR* = 1.61, 95% CI = [1.32,1.95], p = 1.7 × 10^-6^) could predispose to PD (Fig. 3a; Supplementary Table S6). Additionally, increased mean CT may contribute to OCD (*OR* = 1.45, 95% CI = [1.06,2.00], p = 0.02), and reduced ICV may lead to ADHD (*OR* = 0.81, 95% CI = [0.73,0.90], p = 7.0 × 10^-5^). Unlike the forward direction, reverse GSMR analysis did not suggest strong causal effects of disease on the brain (Fig. 3b; Supplementary Table S6). The effect of SZ on reduced SA (*beta* = -0.04, 95% CI = [-0.07,-0.01], p = 5.8 × 10^-3^) implies a potential bidirectional relationship, with a nominal significance that might need further exploration. Study-wise significance of SZ leading to reduced ICV (*beta* = -0.05, 95% CI = [-0.08,-0.02], p = 4.5 × 10^-4^) and the nominal significance of BPD leading to increased total SA (*beta* = 0.09, 95% CI = [0.01,0.17], p = 2 × 10^-2^) was also found. ALS displayed significant effects on lower SA/ICV but the small number of IV SNPs for ALS suggests the necessity for replication to enhance robustness. The robustness of the MR analyses, particularly the forward direction, presented here was substantiated by the other MR methods (Supplementary Table S7; Supplementary Fig. S8; Supplementary Results), as well as MR sensitivity analysis (Supplementary Fig. S9; Supplementary Results).

### 3.4 Clinical diagnoses related to increased genetic risk and a mild reduction in cortical size

**Fig. 4.**
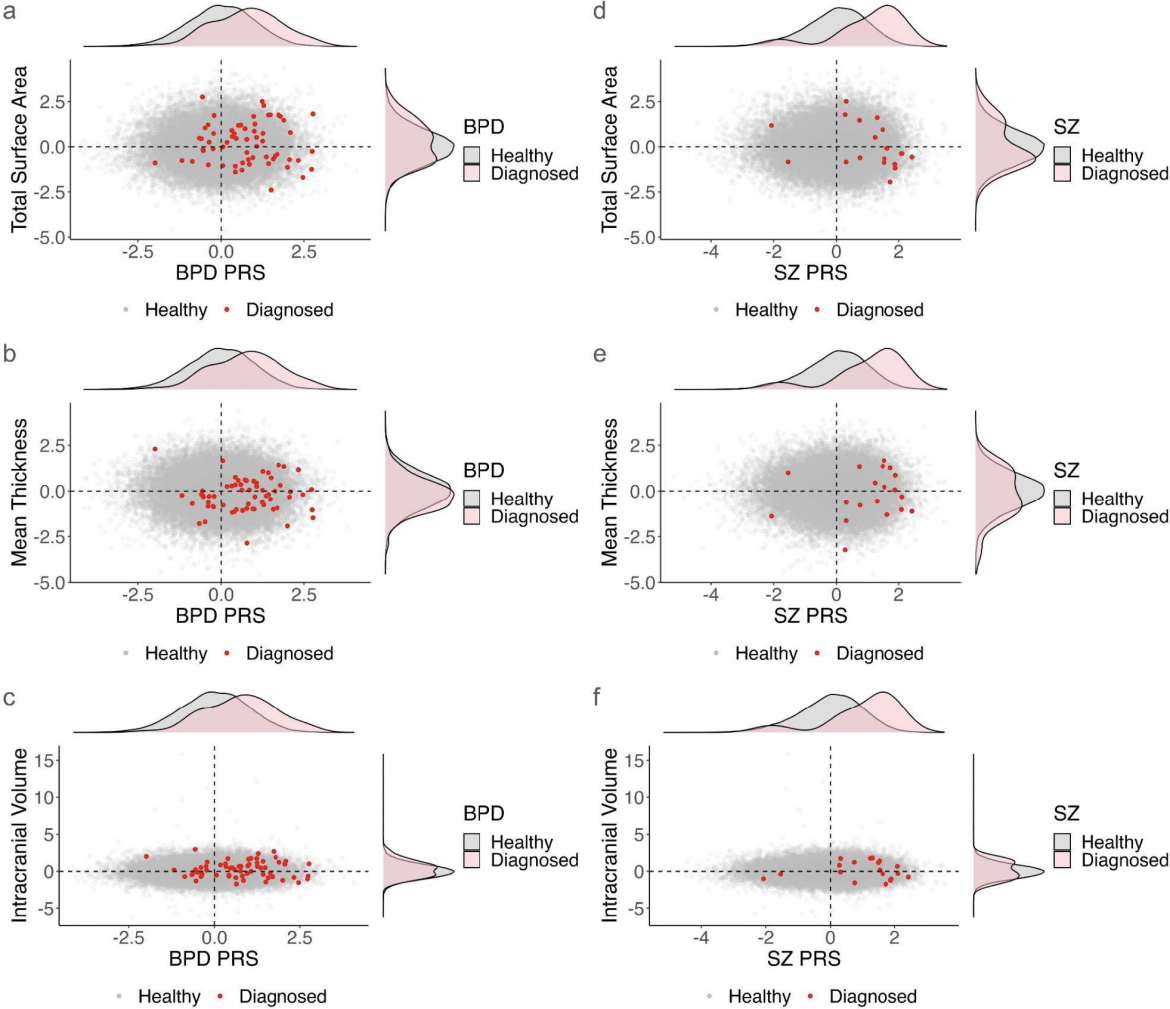
Comparing genetic liability and brain structure distributions between healthy individuals and those with psychiatric conditions. Panels (a) to (f) depict the relationship between standardized brain structure variation and standardized PRS of neuropsychiatric diseases. In the scatterplot, the x-axis represents PRS, and the y-axis reflects brain structure. The marginal density plots, positioned on the top and right of the scatterplot, demonstrate the distributions of PRS and brain structure, respectively. Individuals diagnosed with the corresponding neuropsychiatric diseases are denoted as red dots in scatterplots and pink areas in density plots; healthy individuals are denoted as gray dots and gray areas in density plots. (a, b, c) Total SA, Mean CT, and ICV vs BPD PRS (BPD diagnosed n = 63). (d, e, f) Total SA, Mean CT, and ICV vs SZ PRS (SZ diagnosed n = 19).

Next, we assess whether elevated genetic risk corresponds to clinical diagnosis by mapping patients onto the distribution of genetic liability. We further evaluate their positions within brain structure distributions to characterize the gene–brain–disease relationship. Utilizing data from UKB, we plotted scatterplots to observe the distribution of PRS among diagnosed individuals (Fig. 4). Compared with healthy individuals, there is a shift toward high PRS among the patients. To better observe the difference, we categorized the samples into four groups via sign contingency tables. In Figure 4b, there is a subtle reduction of CT in BPD patients, and the binomial tests reveal statistical significance, indicating enrichment of patients with high PRS and reduced mean CT with p-values of 1.47 × 10^-2^. Figure 4d and 4e focused on SZ, with the binomial tests showing an overrepresentation of patients with decreased SA and CT with p-values of 2.28 × 10^-3^ and 2.87 × 10^-2^. The other panels did not demonstrate significance (Supplementary Table S9).

## 4 Discussion

We observe significant correlations between neuropsychiatric PRS and brain measures. These associations indicate spatial patterns akin to those observed in case–control studies, with small effect sizes as expected in mostly healthy individuals. In other words, even without a formal diagnosis, genetic risk alone influences morphometry, reflecting milder forms of the brain alterations seen in disease in some disorders. Directly comparing the morphometry of genetic risk with diagnoses in a subset of these diseases confirms the patterns observed in previous studies of neuropsychiatric illness (Boedhoe et al., 2017; Caligiore et al., 2016; Hibar et al., 2016, 2018; Hoogman et al., 2017; Logue et al., 2018; Schmaal et al., 2016, 2017; Sun et al., 2022; van Erp et al., 2016, 2018; van Rooij et al., 2018; Walton et al., 2022; Whelan et al., 2018). High polygenic burden corresponds to patient patterns, showing reduced brain volumes and increased ventricles across most disorders. This study replicates associations between increased lateral ventricle volume and SZ (Blokland et al., 2024; Del Re et al., 2019; van Erp et al., 2016), MDD (Schmaal et al., 2016), BPD (Hibar et al., 2016), and ASD (Prigge et al., 2021).

The spatial concordance between genetic risk and disease-related brain changes was assessed using sign concordance and correlation tests. While sign concordance is more easily achieved, both methods consistently identified the same set of disorders, with spatial correlation tests highlighting a smaller subset of diseases (Table 2). Together, high concordance was observed for ADHD, SZ, ASD, and BPD, seemingly for disorders with higher heritability and more neurodevelopmental origins. The effect sizes varied among psychiatric disorders and brain regions. In case–control comparisons from the ENIGMA studies, the larger effect sizes were observed in ADHD and SZ. Subcortical volumes also exhibited high effect sizes across all disorders. The trend is again mirrored in our analysis for high–low PRS comparisons, where ADHD and SZ show higher effect sizes, along with subcortical volumes—evolutionarily conserved regions involved in emotional processing and implicated in various psychiatric disorders.

The smaller effect sizes observed for the genetic-risk brain maps compared with the diagnostic case–control maps in Figure 2 are expected. As discussed in the introduction, this aligns with the view that neuropsychiatric disorders represent the extremes of quantitative traits—such as variations in brain structure—shaped by polygenic predispositions. Accordingly, PRS–brain association effects are attenuated in magnitude; however, the spatial concordance with diagnostic patterns highlights shared underlying brain-morphology signatures that span the continuum of genetic liability.

We presented results from both absolute and relative measures to isolate the genetic influence on specific brain regions. Prior research indicates strong correlations between regional and global measures, particularly in the fronto-dorsal region (Makowski et al., 2023). Adjusting for global effects may weaken regional signals, requiring larger sample sizes for reliability. Thus, in Figure 2, we prioritized absolute measures, which offer better statistical power and the current sample size may fail to detect relative regional effects. However, when absolute measures were unavailable in ENIGMA studies, we used relative measures to ensure alignment with the same modeling approach across analyses.

MR assesses the causal effect of an exposure on an outcome using genetic variants as instruments, similar to randomized controlled trials. The MR estimate reflects the phenotypic effect such as including both genetic and environmental. Among MR studies in neuropsychiatric disorders (Guo et al., 2022; Stauffer et al., 2021; Walton et al., 2019), Guo et al. included several major psychiatric conditions and found evidence of forward causality, brain measures affect disorders (but not the reverse), for SZ, BPD, and AN (Guo et al., 2022). These findings are consistent with our results, which predominantly indicate forward causality.

### 4.1 ADHD

Prior work shows that children with ADHD tend to have lower SA values than control subjects, whereas this trend is not observed in adolescents or adults with ADHD (Hoogman et al., 2019). SA reduction results are widespread, likely attributed to a decrease in total SA. The same pattern has also been identified with genetic risk for ADHD via the PRS analysis here. Notably, unlike other disorders with enlarged ventricles, ADHD genetic risk is associated with smaller ventricles. Moreover, our GSMR analysis provides evidence of a significant causal association between smaller SA/ICV and ADHD, indicating that reduced area and ICV may contribute to the development of ADHD, which is also mentioned in other studies (Ahn et al., 2022). In the subcortical structures, the significant associations align with those previously reported by ENIGMA (Hoogman et al., 2017). These results support the notion that the overall decrease in brain size may play a role in the etiology of ADHD.

### 4.2 SZ

Studies have reported decreased SA and CT in SZ (van Erp et al., 2018), indicating widespread thinner cortex. Specific regional differences emerge in relative regional associations, with smaller cortices in temporal and frontal lobes, and increased size in parietal and paracentral regions in SZ. We also observed these morphological changes associated with SZ PRS, albeit with only nominal significance, except for greater significance found in temporal pole thickness. Thus, we do not observe strong effects of SZ genetic risk associated with a reduced cortex, but smaller total SA is significantly implicated in the development of SZ, as shown in the GSMR analysis here. For the subcortical regions, SZ PRS was linked to increased ventricles. These results broadly align with previous studies of SZ (Franke et al., 2016; Ohi et al., 2020; van Erp et al., 2016), such as higher SZ PRS was associated with smaller frontotemporal cortices (Alnæs et al., 2019; Qi et al., 2022; X. Zhu et al., 2021). A subcortical–PRS association study found no significant associations, except for a notable link with reduced pallidum volume (Grama et al., 2020). However, we did not observe a significant association between SZ PRS and this subcortical structure, although the direction of effects remained consistent.

### 4.3 BPD

Only nominal significance was observed in the PRS–brain association here, although a consistent pattern of increased SA and decreased CT was noted across the cortical absolute measures. Consistently, an overall CT reduction was also observed in patients from the ENIGMA study; however, unlike the increased SA associated with genetic risk of BPD, patients exhibited a mild reduction in SA in most regions (Hibar et al., 2018). While the ENIGMA study does not specifically find increases in the orbitofrontal cortex, our results of a nominal positive association between the orbitofrontal cortex and higher genetic risk of BPD are supported by other smaller studies (Damme et al., 2022; Hibar et al., 2018; Xiao et al., 2020). In the analysis involving healthy and diagnosed UKB participants (Fig. 4), a strong association was revealed showing that patients with high PRS for BPD and SZ exhibit reduced brain structure in total SA and mean CT. Combined with the discovery of the PRS association and the comparison of brain maps, these findings support that alterations of brain structure are indicative not merely of consequences but also of the progression pattern of disorders.

### 4.4 PD

Unlike the other diseases studied here, we found that PD was associated with larger brain morphological measures (except for CT that is known to have an inverse relationship with SA) (Hogstrom et al., 2013). This link to a larger brain is supported by previous large-scale studies. Prior GWAS showed a positive genetic correlation between ICV and PD (Adams et al., 2016), and a recent large imaging study also demonstrated larger ICV in PD (Feldmann et al., 2008; Filippi et al., 2020; García-Marín et al., 2023; Laansma et al., 2021). Additionally, García-Marín et al. demonstrated a shared genetic architecture between PD risk and subcortical brain morphology, particularly ICV and basal ganglia volume. This study identifies overlapping genes involved in autophagy, vesicle trafficking, and neuroinflammatory regulation, suggesting that genetic factors that could contribute to larger early life brain structures may also increase susceptibility to PD later in life (García-Marín et al., 2023). Using PRS, PD genetic risk has been linked to a global increase in SA, consistent with our findings (Abbasi et al., 2022). Other studies show mixed conclusions, presumably due to different stages of the disease. While brain atrophy has been observed, increases in certain regions of SA such as the superior frontal and occipital cortex (Jubault et al., 2011) as well as basal ganglia have also been reported in early stage patients (Reetz et al., 2009). This supports our findings that healthy individuals with high PRS exhibit associations with increased SA and ICV. As the dataset in Laansma et al. consists of patients from all stages, a significant portion is from the early stages, corroborating the explanations that these increases are compensatory responses that might occur in early disease processes (Laansma et al., 2021). However, our GSMR analysis reveals a significant unidirectional causal relationship between increased SA/ICV and PD, implying that these increases might influence the progression of PD, but not the other way around. Furthermore, the inversion polymorphism at 17q21.31 has been linked to both brain volume and PD, which might influence this observed causal association (Bowles et al., 2022; Koolen et al., 2006). However, SNPs at 17q21.31 were excluded during the QC step to account for long-range LD before GSMR and are, therefore, unlikely contributors. Thus, the exact mechanism of the causal relationship remains an open question, prompting the need for further investigation.

### 4.5 AD, PTSD, and OCD

We demonstrated that reduced hippocampal and thalamic volumes, along with enlarged ventricles, are associated with AD genetic risk, aligning with established patterns in AD patients (Aggleton et al., 2016; Apostolova et al., 2012). Complementing our findings, Couvy-Duchesne et al. demonstrated that both polygenic and APOE-ε4 were linked to the same hippocampal and thalamic signatures observed in diagnosed AD (Couvy-Duchesne et al., 2025). Importantly, they manifest that these patterns emerge progressively from MCI converts to AD, reinforcing that the genetic effect on subcortical morphology variation may extend to preclinical markers along AD progression. Smaller ICV is linked to PTSD genetic risk, while larger ICV is associated with PD, mirroring corresponding patterns observed in patients (Laansma et al., 2021; Logue et al., 2018). Findings based on smaller-sample GWAS, such as those on OCD, remain inconclusive and warrant further investigation. However, we replicate ENIGMA’s finding of a significant association with an enlarged pallidum in adults with OCD, a region known to be increased in this condition (Boedhoe et al., 2017). Overall, comparisons with patient patterns indicate an overlap in regions associated with both health and disease states.

## 5 Conclusion

The influence of genetic risk factors for neuropsychiatric disorders on brain structure in the UKB population resembles the brain changes observed in individuals with these disorders, but the effects are of mild magnitude as expected in a largely community-based volunteer population. Neuropsychiatric disorders with relatively high heritability and neurodevelopmental origins were notable in the analyses, demonstrating a marked similarity in brain changes between individuals with clinical diagnoses and those with genetic risk alone for ADHD, SZ, ASD, and BPD. MR analysis uncovers the causal link between reduced SA with SZ and ADHD, supporting that brain structure may be part of the causal pathway of these diseases. In other words, alterations in brain structure may play a role in the etiology of a disease, implying that changes in brain structure may precede or contribute to the onset or manifestation of the disease. Some reverse causality, where the diseases affect the brain, was seen but less significant. Additionally, high genetic risks do not necessarily lead directly to diagnosis, but for some cases, the combination of high genetic risks and smaller brain structures is associated with an increased likelihood of disease occurrence. Other observations that will need to be confirmed are that spatial concordance between genetic risk and disease states in brain changes is higher for young patient groups for SA. For instance, brain regions across SA linked to high PRS for ADHD and MDD resemble those in pediatric ADHD or younger MDD (under 25 years), respectively, but not their adult patients (Supplementary Fig. S3). In contrast, for CT, the opposite pattern is observed, with stronger concordance in adult groups for BPD. Whether these age-specific differences are biological or the result of statistical power differences remains to be determined.

## 6 Limitations

A major limitation of our study is the European ancestry-only inclusion, possibly missing values in generalization for all populations. Differences in the heritability of diseases analyzed, as well as the size of the GWAS, might affect the statistical power. High polygenicity of certain disorders, such as MDD, further impacts results (Ge et al., 2019). Here, our focus is limited to the gray matter of cortical structure and subcortical volume, excluding the examination of other informative structural and functional features such as those derived from diffusion and functional MRI. Future studies can incorporate these additional features. Some disorders from ENIGMA provide only global unadjusted (absolute) or adjusted (relative) regional measures. Having both types of measures would be ideal for a more thorough analysis. There is a scarcity of large-scale GWAS data on age-stratified studies of neuropsychiatric disorders. As a result, this study is limited in its ability to address potential differences between pediatric and adult cohorts. Future research may explore these age-stratified variations in greater detail. Another methodological consideration is the use of a median split for PRS dichotomization. Although this approach was selected for interpretability and balanced for group sizes, dichotomizing a continuous measure can reduce statistical power. Future work could incorporate percentile-based (e.g., top 10% vs bottom 50%) or continuous PRS modeling to optimize power and contrast while maintaining compatibility with case–control data. One limitation of the sex-specific analysis is insufficient statistical power given the current sample size. Additionally, the lack of sex-stratified GWAS summary data for neuropsychiatric conditions precludes MR analysis to infer causality. For instance, while females with BPD risk exhibited associations with reduced CT, particularly in the dorsolateral prefrontal cortex, causal relationships could not be investigated. Addressing these limitations will require larger sample sizes and access to sex-stratified GWAS summary statistics for more robust and causal insights.

## Supplementary Material

Supplementary Material

Supplementary Tables

## Data Availability

The individual-level genetic and neuroimaging data used in this study were obtained from the UK Biobank (https://www.ukbiobank.ac.uk/). UK Biobank data are available to approved researchers. Summary-level neuroimaging results for neuropsychiatric disorders were obtained from published studies conducted by the ENIGMA consortium and related ENIGMA working groups, which are publicly available through the ENIGMA consortium and associated publications. Genome-wide association study (GWAS) summary statistics used for polygenic risk score construction were obtained from publicly available sources, as detailed in Supplementary Table S1. All analyses were conducted using publicly available software, including FreeSurfer for neuroimaging processing, PRS-CS for polygenic risk score estimation (https://github.com/getian107/PRScs), GCTA for genetic analyses (https://yanglab.westlake.edu.cn/software/gcta/#Overview), and GSMR for Mendelian randomization analyses (https://github.com/JianYang-Lab/gsmr/releases). Custom scripts used for data processing and statistical analyses are available upon request.
