# Supplementary material for: Brain patterns linked to neuropsychiatric genetic risk mirror those seen in disease
Source: Imaging Neurosci (Camb). 2026 Mar 10;4:IMAG.a.1152. doi: 10.1162/IMAG.a.1152 (PMC12977089; doi:10.1162/IMAG.a.1152)
Supplement: Supplementary Material [file IMAG.a.1152_supp.pdf]

Supplementary Information

Table of Contents

Supplementary Methods.
Supplementary Figures.
SFigure 1. Normal distribution of PRS for neuropsychiatric diseases.
SFigure 2. Heatmap of associations between polygenic risk scores and all subcortical regions.
SFigure 3. Heatmap of association between ADHD PRS and cortical surface area, cortical thickness across childhood and adulthood cohorts.
SFigure 4. Heatmap of sex differences in associations between polygenic risk score and cortical/subcortical absolute measurements.
SFigure 5. Cohen's d brain maps illustrate the differences in cortical measurements between diagnosed versus healthy controls, as well as high and low-risk scores for neuropsychiatric disorders.
SFigure 6. Comparison in Cohen's d brain maps between diagnosed age groups in the cortical surface area measurements of ADHD, BPD, OCD, and MDD.
SFigure 7. Comparison in Cohen's d brain maps between diagnosed age groups in the cortical thickness measurements of ADHD, BPD, OCD, and MDD.
SFigure 8. Heatmap of causal effects of global measures (total surface area, mean thickness and ICV) on the polygenic risk scores of 13 neuropsychiatric phenotypes.
SFigure 9. Funnel plots and Leave-one-out plots of the effect of global measures (total surface area, mean thickness and ICV) on the polygenic risk scores of statistically significant pairs from MR.
Supplementary Reference.

This supplemental material has been provided by the authors to give readers additional information about the work.

Supplementary Methods.

1. Details on determining the t_e for independent phenotypes

In our case, the analysis of 26 cortical phenotypes comprises regional measures (12 SA, 12 CT averaged across hemispheres) and global measures (total SA, mean CT). Therefore, we used a significance threshold of P<2.3e-3 (0.05/t_e, with t_e=22 being the effective number of independent traits). [1] For the 8 subcortical regions with one global measure of ICV, the estimated t_e is 7.

2. Details on sample description for age-stratified cohort

In order to investigate the PRS association with cortical measurements in different developmental stages, we included GWAS summary statistics for age-stratified cohorts.[2] The cohorts included childhood group (N = 14,878) and late-diagnosed (N = 6961), with control participants (N = 38,303). The PRS calculation followed the same process pipeline and quality control outlined in the method section in the main manuscript.

3. Investigating sex differences in PRS and brain measurement associations

To examine sex differences in the associations between PRS and brain measurements, we conducted interaction analyses involving PRS of neuropsychiatric disorders. The study focused on the potential modifying effect of sex by including an interaction term (sex × PRS) in linear regression models. For diseases showing nominal significance in more than one region, post-hoc analyses were subsequently conducted for males and females separately. These analyses focused on examining the directionality and magnitude of effect sizes for those specific associations only. Significant interaction terms (cortical: $p < 2.3\times10^{-3}$, subcortical: $p < 7.2\times10^{-3}$) highlighted sex-specific associations.

4. Details on comparative analysis contrasting brain maps associated with disease states and genetic risk

To evaluate the agreement between diagnosis and genetic risk between these two brain maps, we computed the proportion of brain regions showing consistent effect size signs between them, in addition to significant correlations.

The agreement of effect size was additionally validated using the sign concordance test, correlation test, and permutation test. The sign concordance test results assessed the sign concordance of effect sizes between ENIGMA case-control data and our high vs. low PRS analysis through a binomial test where both signs surpass the chance expectation with a significant threshold of $p < 0.05$. A correlation test was conducted with the cor.test() function in R to compute the correlation for the effect size values between ENIGMA and our PRS analyses. Due to spatial dependence among brain regions, the significance of the correlation was further evaluated through the permutation test. It assessed the significance of the correlation between two effect size values through reshuffling, comparing the observed correlation to a null distribution based on the reshuffling of values within one group (10,000 times without replacement). The p values from both correlation and permutation tests were consistent. Results are detailed in the supplementary section. (STable8).

5. Details on the methods for Mendelian randomization and sensitivity analyses

To derive candidate instrumental variables (IVs), SNPs associated with the brain measures were extracted from brain GWAS for forward MR analysis, while SNPs associated with disorders were extracted from disease GWAS for the reverse MR analysis. Independent SNPs were selected via the PLINK clumping feature with a threshold of significant SNPs ($p < 5\times10^{-8}$), linkage disequilibrium (LD)-independent ($r^2<0.001$, 1Mb) with the LD reference from the UKB data.

In line with QC workflow[3], we implemented the following steps. SNPs in the long-range LD were excluded based on the GWAS summary. [4] Subsequently, SNPs associated with potential confounder factors—including education level, drinking and smoking behavior, and socioeconomic status—were removed based on the traits, that are known to alter brain structure.[3] To mitigate the pleiotropic effect, SNPs that are directly or indirectly associated (via LD) with the disease were identified as the significant SNPs present in the disease GWAS ($p < 5\times10^{-8}$, LD $r^2 > 0.8$, 1Mb), and were subsequently removed.

For the case-control GWAS, the odds ratio (OR) was converted to beta via logOR. The pipeline in the TwoSampleMR v0.5.8 R package was conducted to remove the palindromic SNPs with minor allele frequencies close to 0.5.

Further MR analyses were performed through various methods such as weighted median, inverse-variance weighted (IVW), Egger regression, and weighted mode utilizing the TwoSampleMR v0.5.8 R package. Individual disease results with a minimum of 3 SNPs, MR sensitivity analysis was conducted through the functions mr_singlesnp() and mr_funnel_plot() to generate funnel plots; mr_presso() to check horizontal pleiotropy and outlier; also, the mr_leaveoneout() and mr_leaveoneout_plot() to generate LOO plots.

Generalized summary-based MR (GSMR) was conducted with the gsmr v1.1.0 R packages.[5] We followed the standard threshold of the package and implemented it with a reference sample size of 8000, a minimum number of instruments of 3, and the HEIDI method to exclude the pleiotropic effect in the analysis.

6. **Details on comparing scatterplots and density distributions of PRS and brain structure between healthy and diagnosed individuals.**

After acquiring the dataset comprising 33860 participants from UKB, we selected the diagnosed individuals with specific neuropsychiatric diseases for further statistical analysis. This subset included BPD (N=63) and SZ (N=19). Before analysis, we controlled for the confounding factors by regressing the global structure measurements against age, sex, scanner site, a proxy of scan quality (FreeSurfer´s Euler number)[6], and the first ten genetic principal components. The residuals are obtained and rescaled via centering and applying standard deviation units. Similarly, PRS values were rescaled through the same methods. Subsequently, the standardized global measurement residuals and PRS values were categorized into four groups using sign contingency tables: both positive PRS and brain measure, both negative PRS and brain measure, positive PRS with negative brain measure, and negative PRS with positive brain measure. We hypothesize that both high genetic liability and smaller brain sizes are risk factors for psychiatric conditions. The null hypothesis posits that the proportion of patients exhibiting positive PRS and negative brain measures is equal to 0.25. Binomial tests were conducted to assess the diagnosed individuals with positive PRS and having reduced global brain measurements significantly differ from the random chance of 25%.

7. Details on clinical diagnosis for mental disorders in the UKB

We defined SZ and BPD in UKB as diagnoses reported in field 130874 (Date F20 first reported, schizophrenia) and 130892 (Date F31 first reported, bipolar affective disorder). These data fields include diagnoses if present in at least one of: self-report (field IDs 20008/20544), ICD-10 medical record diagnosis from hospital admissions (field IDs 41270/41202/41204) and death records (field IDs 40001/40002), or an equivalent read code from primary care records (field ID 42040). Full descriptions of these sources are available on the UKB Showcase[7].

Self-Reported Data. Participants were asked about a SZ or BPD diagnosis on two distinct occasions: initially during the recruitment interview and later in an online Mental Health Questionnaire (MHQ)[8]. During initial recruitment interviews participants were asked if they had been informed by a doctor about having any serious illnesses or disabilities. There was no direct prompt for a SZ or BPD diagnosis; however, if participants disclosed it, this information was recorded. Invitations to complete the online MHQ were emailed in 2016/2017 to 333,420 participants. A total of 157,328 participants completed the MHQ. The questionnaire asked, "Have you ever been diagnosed with one or more of the following mental health problems by a professional, even if you don't have it currently?" Among the conditions listed were SZ, psychotic disorders, mania, hypomania, bipolar or manic depression[8]. For additional details on these responses, refer to UKB Fields 20002 and 20544.

Hospital admission records are available separately for England, Wales, and Scotland from their respective National databases. These records date back to 1997 for England, 1998 for Wales, and 1981 for Scotland. NHS hospital care records were available for all participants who have been admitted to the hospitals during the coverage period. More details on hospital admissions can be found in UKB Resource 138483[9].

Data on deceased UKB participants is provided by NHS England for those in England and Wales and by the NHS Central Register (NHSCR), part of the National Records of Scotland, for participants in Scotland. This mortality data is updated quarterly and sent to the UKB. Further details can be found in UKB Resource 115559[10].

At the time of analysis, primary care data was available for approximately 45% of participants in the UKB study. The UK does not have a centralized system for collecting or sharing primary care data. Additional details can be referenced in UKB Resource 591[11].

Supplementary Results.

1. **Comparative analysis contrasting brain maps associated with disease states and genetic risk in subcortical volume**

The comparison findings for subcortical volume are demonstrated in Supplementary Figure 5. (STable 8) Subcortical volumes also exhibited high effect sizes across all disorders. The trend from the case-control studies is again mirrored in our analysis for high-low PRS comparisons. It is noteworthy that, within the limited regions provided, there is an overlap in 5 out of 8 regions for ADHD. This similarity endures despite the evident milder presentation of abnormalities within the genetic risk group. For the relative regions, the results reveal that pediatric patients exhibit a disproportionately smaller frontal area in relation to the total brain area when compared to the control group. This suggests that a specific reduction in frontal brain region size is observed in the patient population after accounting for global variations in brain measurements.

2. **Cortical morphology associated with polygenic risks of ADHD for age-stratified cohort**

In the analysis, we explored the associations between cortical measurements and PRS for ADHD in two age cohorts: childhood and adulthood. We observed a similar trend of strong negative associations between SA and PRS in the absolute regional cortical measurements. (SFig. 3a) The similarities across age cohorts demonstrate a stable PRS influence on the cortical SA, additionally, no significant associations and variations were found in CT regardless of the age cohorts. These consistent findings suggest that the effects remain stable across different developmental stages. When controlling for global, the relative regional associations showed some nominal significance of PRS on regional CT in the adulthood cohort. (SFig. 3b) However, these findings are limited by the smaller sample size of the adulthood (N = 6961) and childhood group (N = 14,878) compared to the full cohorts (N = 38,691), with the global adjustment making regional association harder to

detect and substantiate. Further research would require a larger sample size to interpret better and understand the variation of different developmental stages.

3. Sex-specific effects on the association between brain morphology and polygenic risk scores of neuropsychiatric disorders

SFigures 4a and 4c present the interaction effects of sex and PRS on brain regions, revealing no significant findings for most disorders (see STable 10). This outcome aligns with the difficulty in detecting interaction effects. However, for BPD, a consistent pattern of nominal significance emerged across several measures, including SA, CT, and ICV. Post-hoc sex-stratified analysis (SFig. 4b and 4d) showed notable differences in PRS-brain associations between males and females (STable 11). These findings emphasize the need to reexamine sex effects in BPD as sample sizes increase.

4. Mendelian randomization methods and sensitivity analysis supporting the consistency and robustness of GSMR discovery

Due to the unclear nature of any observed associations between the brain and neuropsychiatric genetic risk, which may reflect pleiotropy, we also conducted MR and GSMR. The MR estimate reflects the phenotypic effect (both genetic and environmental), despite efforts to minimize the effect by removing confounding factors. We investigated bidirectional causal relationships for the effects of the brain on disease and vice versa.

While most results did not suggest strong causal effects of disease on the brain in reverse (STable 6), the SA on SZ risk scores implies a potential bidirectional relationship and exceptionally more IVs had passed the filter.

The robustness of the MR analyses presented here was supported by the other MR methods Other MR methods including weighted median, inverse-variance weighted (IVW), Egger regression, and weighted mode are implemented to support the GSMR results (STable 6; SFig. 8), as well as funnel plots and Leave-One-Out (LOO) plots for statistically significant MR pairs are provided (SFig. 9). The funnel plots display overall even distributed data points on both sides of the average effect size, indicating minimal bias. Asymmetries in some plots suggest potential heterogeneity but are

further confirmed by the LOO plots that no individual SNP dramatically influences the average effect, implying observed outliers might be due to random variations from the limited SNP sample size. Overall, the findings exhibit the robustness of the MR analyses.

Supplementary Figures.

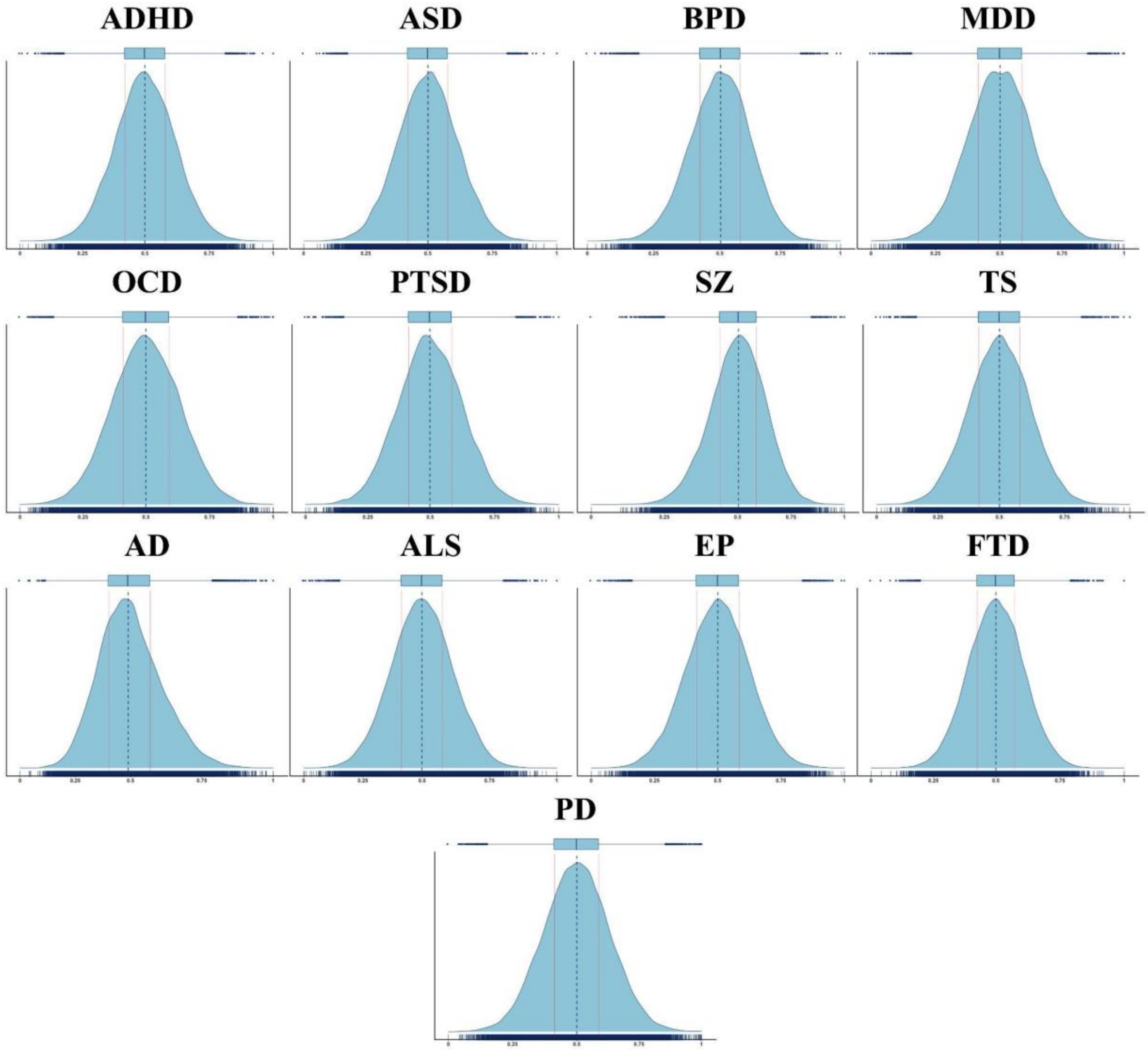

Supplementary Figure 1. Normal distribution of PRS for neuropsychiatric diseases. PRS distribution for Attention-deficit/hyperactivity disorder (ADHD), Autism spectrum disorder (ASD), Bipolar disorder (BPD), Major depressive disorder (MDD), Obsessive-compulsive disorder (OCD), Post-traumatic stress disorder (PTSD), Schizophrenia (SZ), Tourette syndrome (TS), Alzheimer's disease (AD), Lou Gehrig's disease (ALS), Epilepsy (EP), Frontotemporal dementia (FTD), Parkinson's disease (PD) is displayed with density plot. PRS values were rescaled between zero and one without standardization.

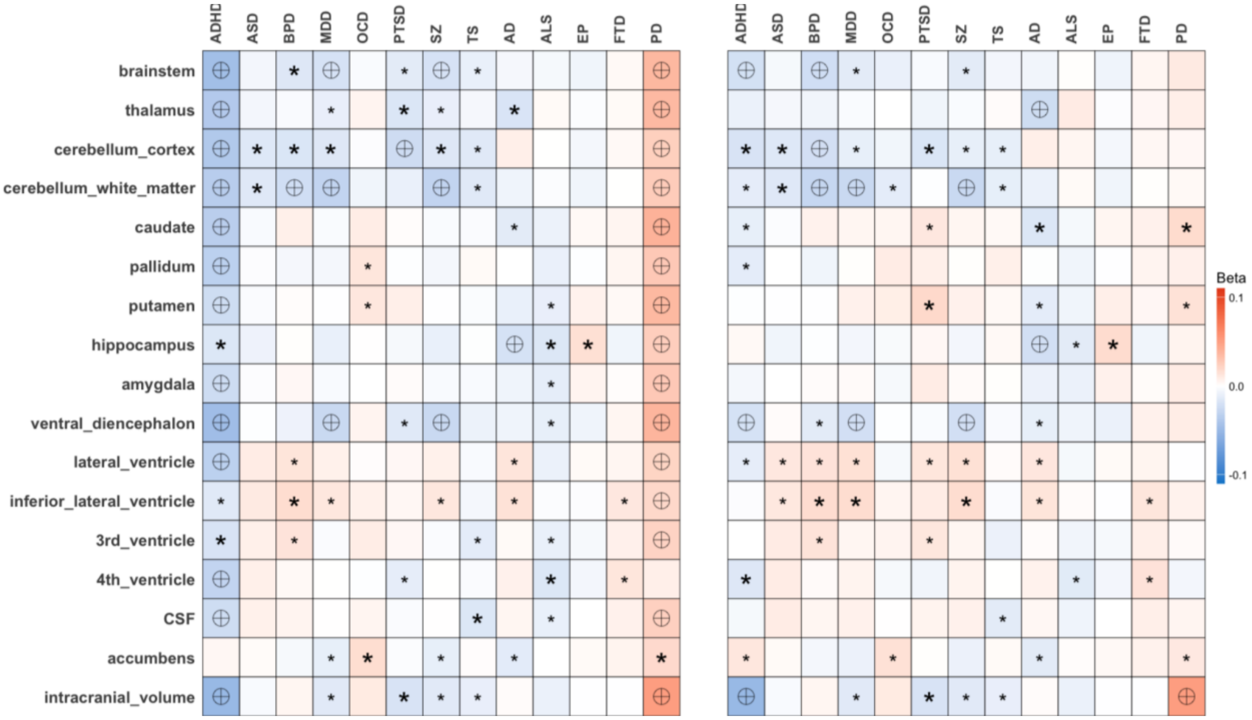

Supplementary Figure 2. Heatmap of associations between polygenic risk scores and all subcortical regions. subcortical measurements for absolute regions (left), and subcortical measurements for relative regions (right). Boxes are colored according to the beta coefficients of linear regression models. Various stringency thresholds are used, including nominal significance ($p < 0.05$, denoted by small asterisks), Bonferroni-corrected threshold for individual diseases (disease-wise significance denoted by large asterisks, subcortical: $p < 4.5x10-3$) and Bonferroni correction for total comparisons (study-wise significance represented by encircled crosses, subcortical: $p < 3.5x10-4$, 11 regions x 13 diseases). This multiple-threshold approach enables us to compare with published studies focusing on single diseases.

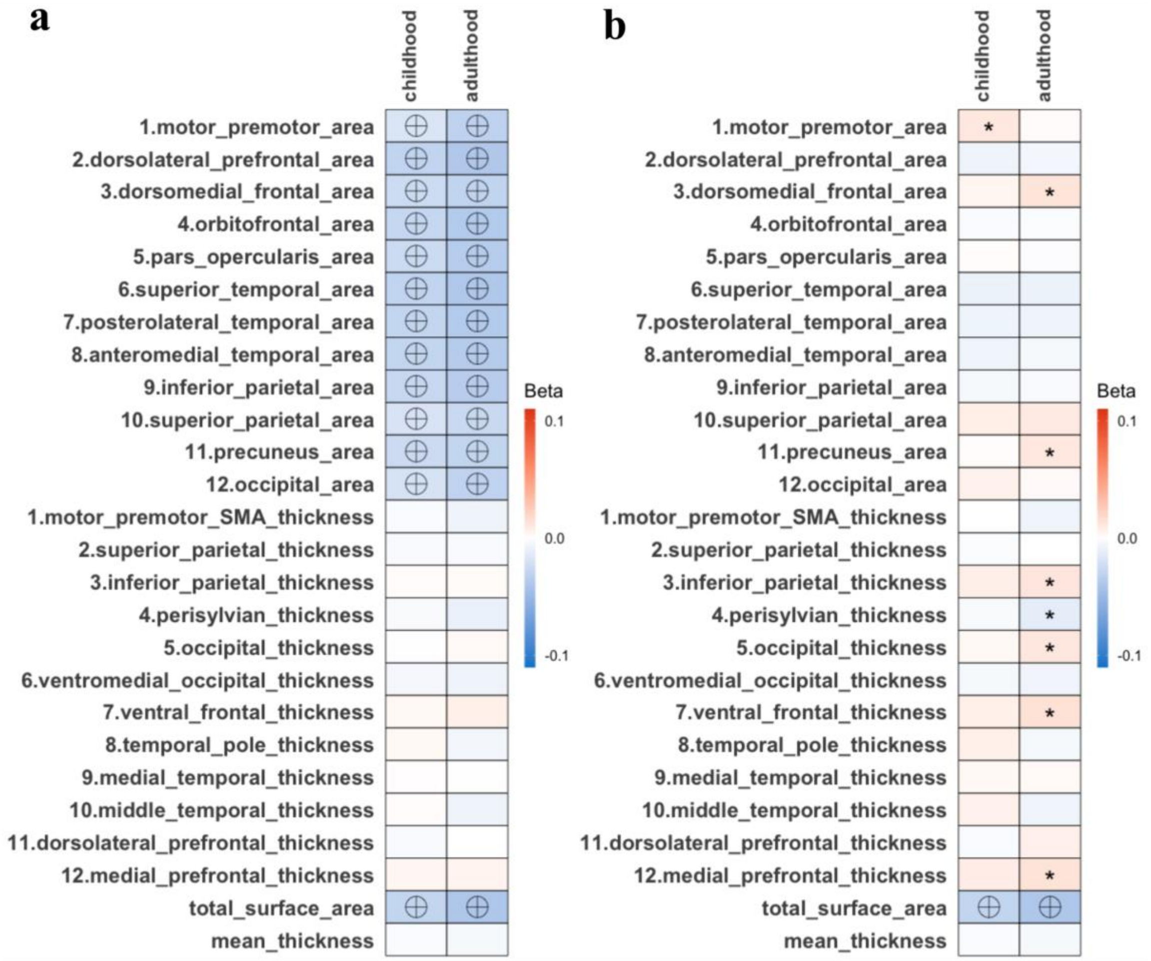

Supplementary Figure 3. Heatmap of association between ADHD PRS and cortical surface area, cortical thickness across childhood and adulthood cohorts. Association between ADHD PRS and cortical surface area for childhood and adulthood,[2] with cortical measurements shown in absolute regions (a) and in relative regions (b). Global measures are presented twice at the bottom of both top heatmaps. Boxes are colored according to the beta coefficients of linear regression models. Various stringency thresholds are used, including nominal significance ($p < 0.05$, denoted by small asterisks), Bonferroni-corrected threshold for individual cohorts (cohort-wise significance denoted by large asterisks, cortical: $p < 2.5\text{x}10^{-2}$) and Bonferroni correction for total comparisons (study-wise significance represented by encircled crosses, cortical: $p < 1.1\text{x}10^{-3}$, 22 regions x 2 cohorts).

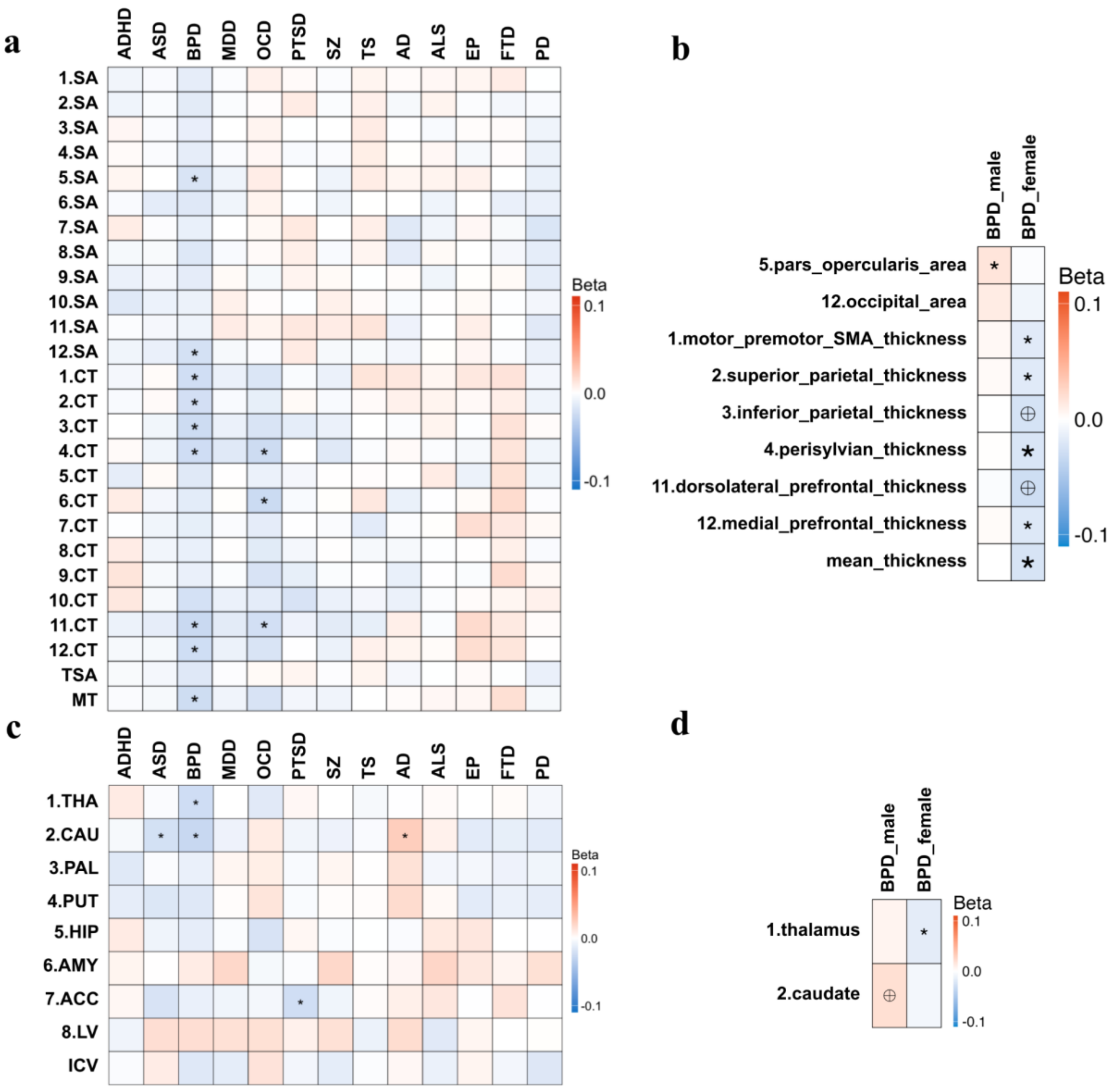

Supplementary Figure 4. Heatmap of sex differences in associations between polygenic risk score and cortical/subcortical absolute measurements. (a) Interaction effects between sex and PRS for 13 disorders for cortical absolute measurements. Post-hoc analysis for (b) BPD sex-stratified association between PRS and cortical measurement. (c) Interaction effects between sex and PRS for 13 disorders for subcortical absolute measurements. Post-hoc analysis for (d) BPD sex-stratified association between PRS and subcortical measurement. For (a)(c) various stringency thresholds are used, including nominal significance ($p < 0.05$, denoted by small asterisks), Bonferroni-corrected threshold for individual diseases (disease-wise significance denoted by large asterisks, cortical: $p < 2.3\text{x}10^{-3}$, subcortical: $p < 7.2\text{x}10^{-3}$) and Bonferroni correction for total comparisons (study-wise significance represented by encircled crosses, cortical: $p < 1.7\text{x}10^{-4}$, 22 regions x 13 diseases, subcortical: $p < 5.5\text{x}10^{-4}$, 7 regions x 13 diseases). For (b)(d) various

stringency thresholds are also used, including nominal significance ($p < 0.05$, denoted by small asterisks), Bonferroni-corrected threshold for individual diseases (disease-wise significance denoted by large asterisks, cortical: $p < 5.6\times10^{-3}$, subcortical: $p < 2.5\times10^{-2}$) and Bonferroni correction for total comparisons (study-wise significance represented by encircled crosses, cortical: $p < 2.8\times10^{-3}$, 9 regions x 2 diseases, subcortical: $p < 1.3\times10^{-2}$, 2 regions x 2 diseases). All regions shown in panel (a)(b)(c)(d) are correspond to the regions labeled in Figure 1.

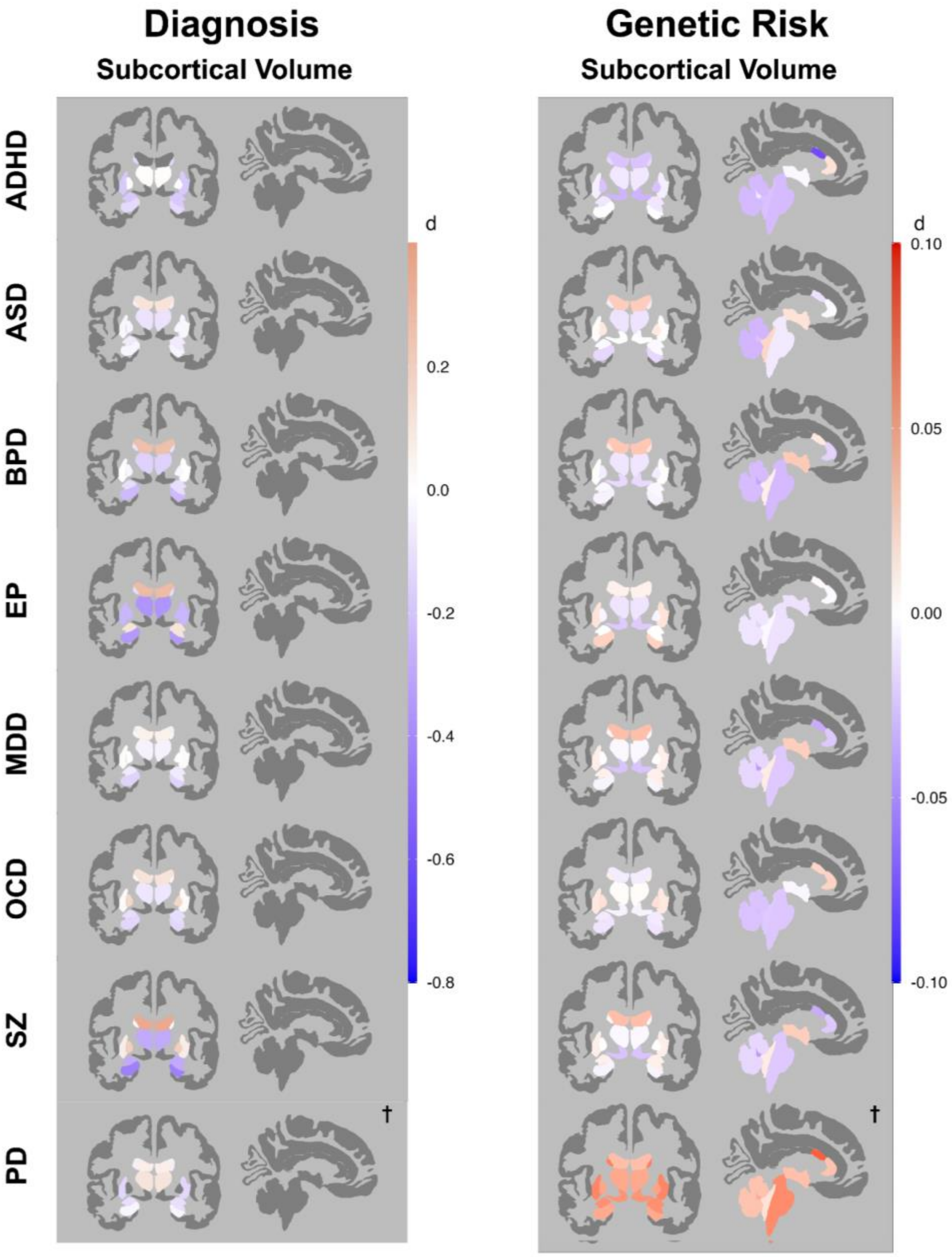

Supplementary Figure 5. Cohen's d brain maps illustrate the differences in cortical measurements between diagnosed versus healthy controls, as well as high and low-risk scores for neuropsychiatric disorders. This figure presents the spatial layouts of subcortical volume, referencing the ENIGMA consortium, for seven disorders: ADHD, ASD, BPD, EP, MDD, OCD, SZ, and PD. On the left panel, it displays the effect size of diagnose measurements referencing from ENIGMA. The right panel represents the effect size of the calculated PRS. Each row demonstrates the morphometric measures' effect sizes by Cohen's d. Color intensity reflects the magnitude of effect size (d), with blue denoting a negative effect and red a positive effect. The color scale for diagnosis is (-0.45,2), and the genetic risk score is (-0.1,0.1). Brain maps accompanied by a cross symbol (†) indicate absolute regional measurements.

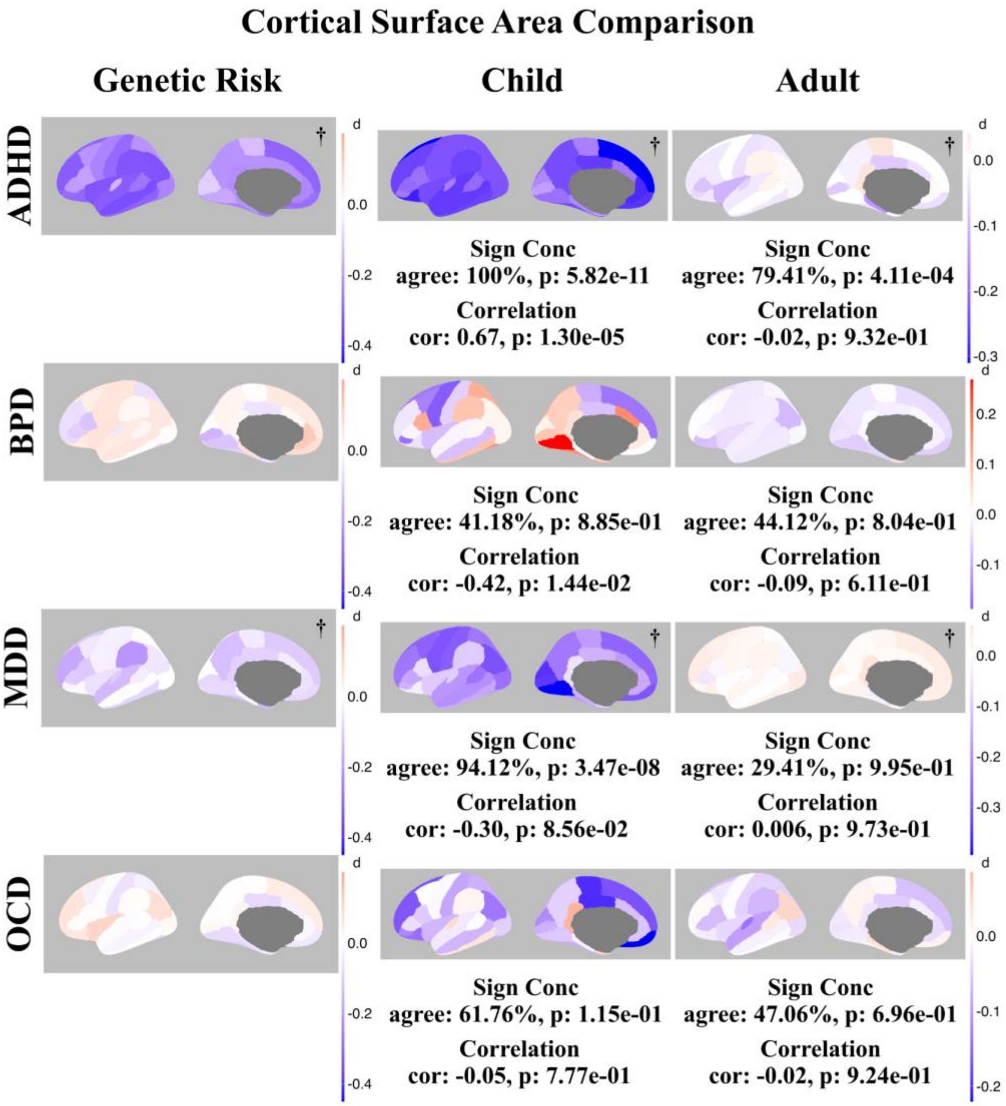

Supplementary Figure 6. Comparison in Cohen's d brain maps between diagnosed age groups in the cortical surface area measurements of ADHD, BPD, OCD, and MDD. This figure presents the spatial layouts of cortical surface area, referencing the ENIGMA consortium, for ADHD, MDD, BPD, and OCD for different age groups. The left panel represents the effect size of the calculated PRS. On the right panel, it displays the effect size of diagnose measurements referencing ENIGMA with different age groups. Each row demonstrates the effect sizes of the morphometric measures using Cohen's d. Color intensity reflects the magnitude of effect size (d), with blue denoting a negative effect and red a positive effect. The color scale for diagnosis adapts to the data range of both age cohorts, and the genetic risk score is (-0.1,0.1). Disorder comparisons are present in absolute and relative regions based on availability and marked as asterisks at the bottom of the layout. Sign concordance and correlation results are displayed beneath the brain maps.Brain maps accompanied by a cross symbol (†) indicate absolute regional measures.

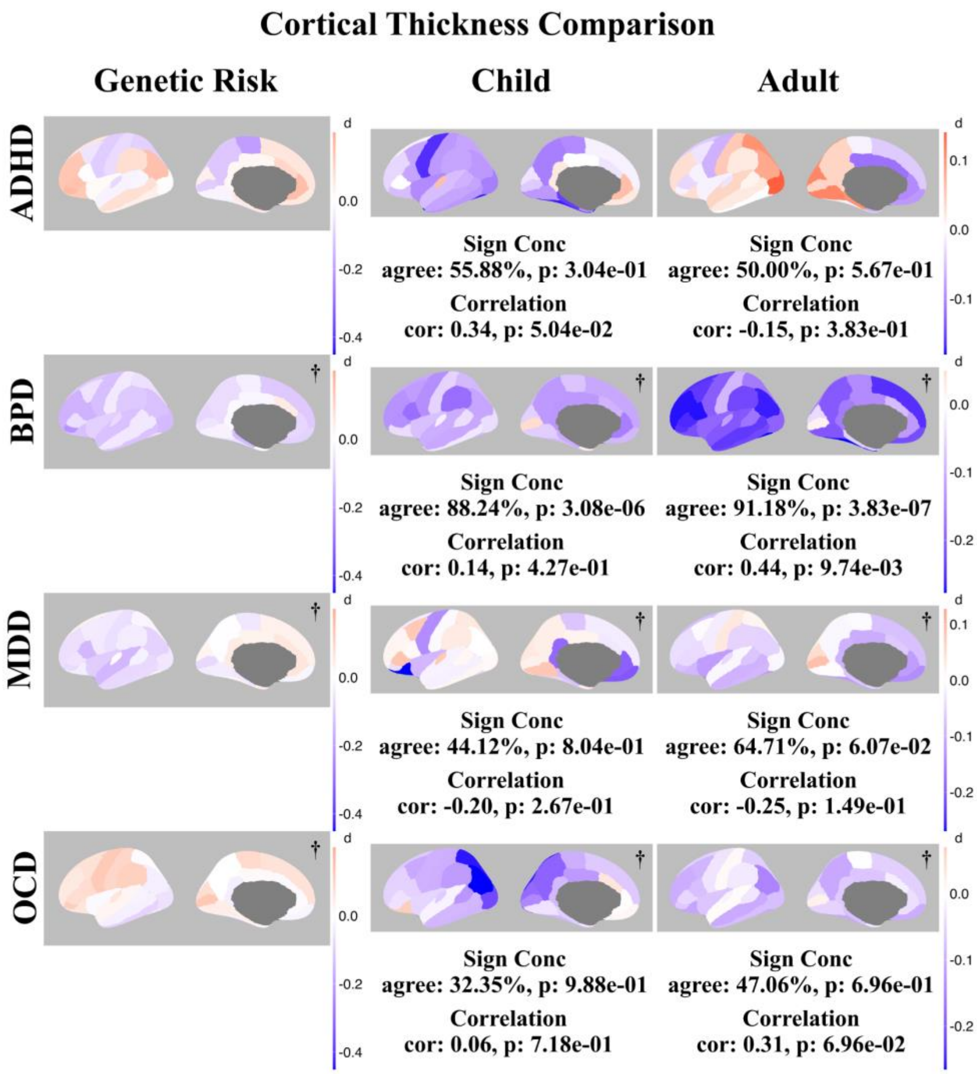

Supplementary Figure 7. Comparison in Cohen's d brain maps between diagnosed age groups in the cortical thickness measurements of ADHD, BPD, OCD, and MDD. This figure presents the spatial layouts of cortical thickness, referencing the ENIGMA consortium, for ADHD, MDD, BPD, and OCD for different age groups. The left panel represents the effect size of the calculated PRS. On the right panel, it displays the effect size of diagnose measurements referencing ENIGMA with different age groups. Each row demonstrates the effect sizes of the morphometric measures using Cohen's d. Color intensity reflects the magnitude of effect size (d), with blue denoting a negative effect and red a positive effect. The color scale for diagnosis adapts to the data range of both age cohorts, and the genetic risk score is (-0.1,0.1). Disorder comparisons are present in absolute and relative regions based on availability and marked as asterisks at the bottom of the layout. Sign concordance and correlation results are displayed beneath the brain maps. Brain maps accompanied by a cross symbol (†) indicate absolute regional measures.

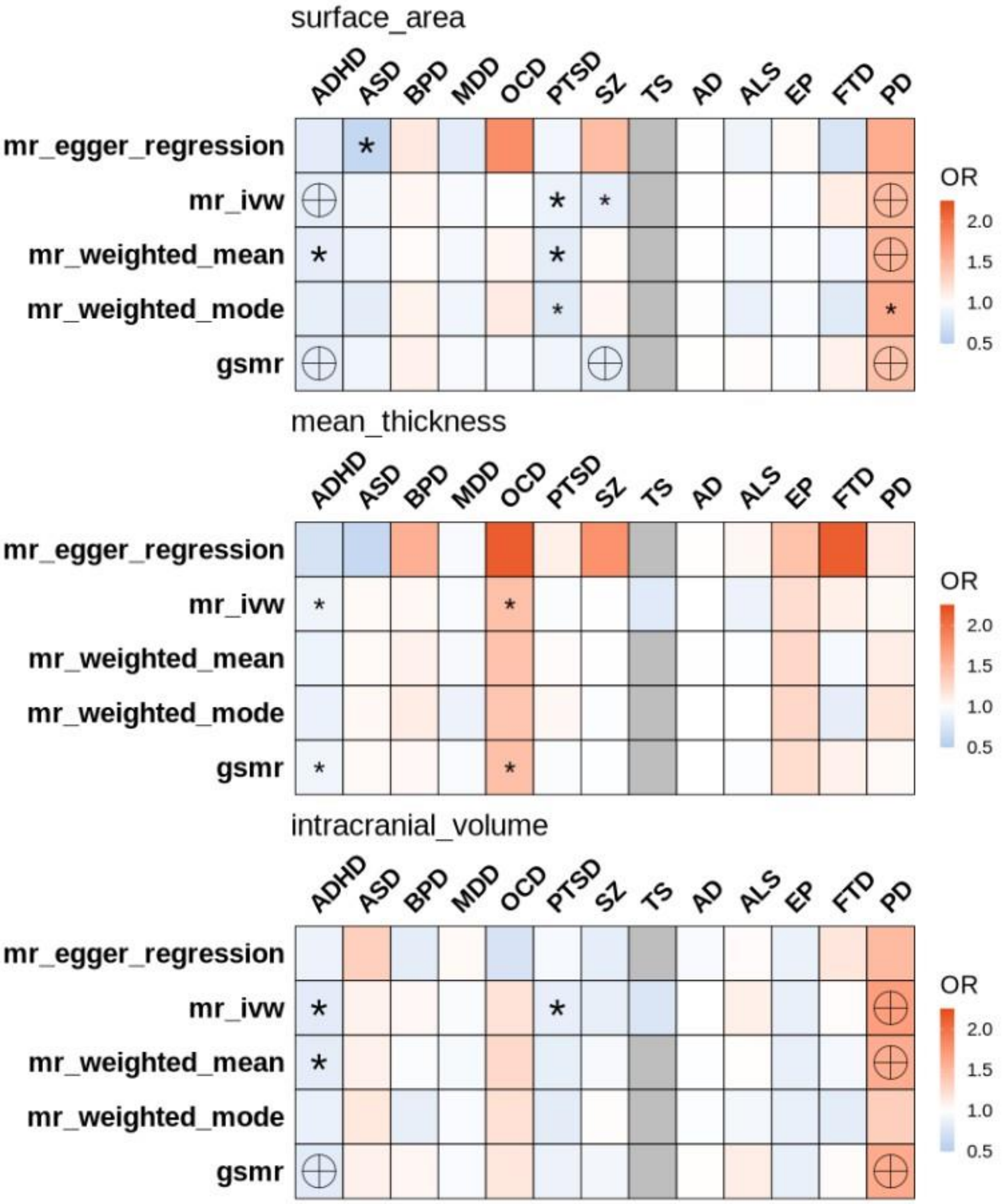

Supplementary Figure 8. Heatmap of causal effects of global measures (total surface area, mean thickness and ICV) on the polygenic risk scores of 13 neuropsychiatric phenotypes. Boxes are colored according to the odds ratio (OR) of multiple unidirectional MR-analysis, including weighted median, inverse-variance weighted (IVW), Egger regression, weighted mode, and GSMR. Labels denote nominal significance ($p < 0.05$, small asterisks), Bonferroni-corrected disease-wise significance ($p < 1.6e-2$, big asterisks), and study-wise significance ($p < 1.2e-3$, encircled crosses). Gray boxes represent data that is not available, attributed to limitations such as insufficient sample size or an inadequate number of filtered SNPs meeting the criteria for inclusion. The same color scale was provided (0.5,2.25).

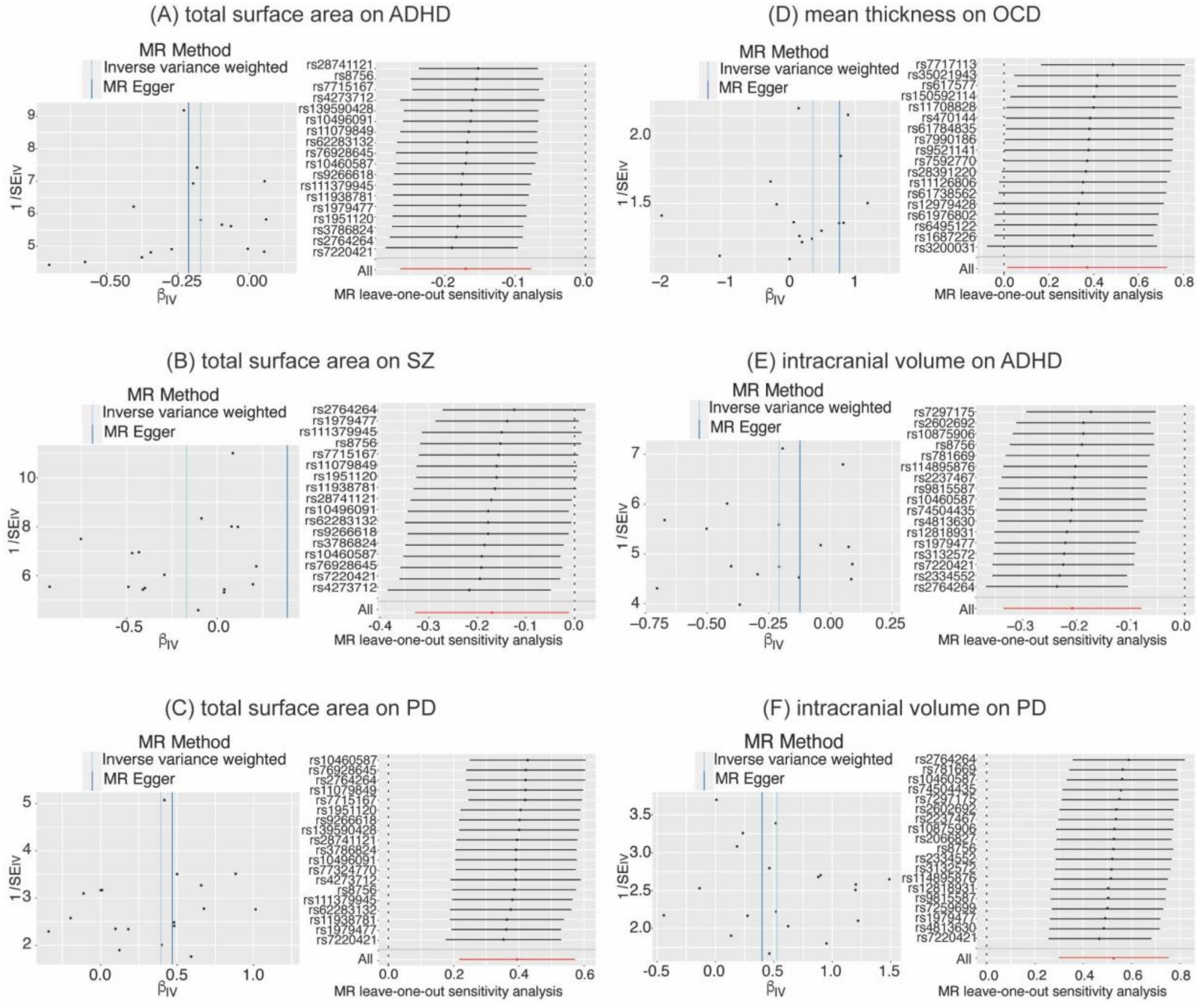

Supplementary Figure 9. Funnel plots and Leave-one-out plots of the effect of global measures (total surface area, mean thickness and ICV) on the polygenic risk scores of statistically significant pairs from MR. Funnel plot: each black data point represents an SNP, plotted by the estimated causal effect of global measurements on the polygenic risk score on the horizontal axis, and the inverse of the standard error of the estimate on the vertical axis. Leave-one-out analysis: each row of solid lines represents the range of estimated effect size of the MR after excluding the corresponding SNP.

Supplementary Reference.

1. Li, M.-X., Yeung, J. M. Y., Cherny, S. S. & Sham, P. C. Evaluating the effective numbers of independent tests and significant p-value thresholds in commercial genotyping arrays and public imputation reference datasets. *Hum. Genet.* **131**, 747–756 (2012).
2. Rajagopal, V. M. *et al.* Differences in the genetic architecture of common and rare variants in childhood, persistent and late-diagnosed attention-deficit hyperactivity disorder. *Nat. Genet.* **54**, 1117–1124 (2022).
3. Guo, J. *et al.* Mendelian randomization analyses support causal relationships between brain imaging-derived phenotypes and risk of psychiatric disorders. *Nat. Neurosci.* **25**, 1519–1527 (2022).
4. Long-Range LD Can Confound Genome Scans in Admixed Populations. *Am. J. Hum. Genet.* **83**, 132–135 (2008).
5. Zhu, Z. *et al.* Causal associations between risk factors and common diseases inferred from GWAS summary data. *Nat. Commun.* **9**, 224 (2018).
6. Dale, A. M., Fischl, B. & Sereno, M. I. Cortical surface-based analysis. I. Segmentation and surface reconstruction. *Neuroimage* **9**, 179–194 (1999).
7. : Showcase Homepage. https://biobank.ndph.ox.ac.uk/ukb/.
8. Davis, K. A. S. *et al.* Mental health in UK Biobank - development, implementation and results from an online questionnaire completed by 157 366 participants: a reanalysis. *BJPsych Open* **6**, e18 (2020).
9. : Resource 138483. https://biobank.ndph.ox.ac.uk/showcase/refer.cgi?id=138483.
10. : Resource 115559. https://biobank.ndph.ox.ac.uk/showcase/refer.cgi?id=115559.
11. : Resource 591. https://biobank.ndph.ox.ac.uk/showcase/refer.cgi?id=591.
